# Probabilistic Inference of Transcription Factor Binding from Multiple Data Sources

**DOI:** 10.1371/journal.pone.0001820

**Published:** 2008-03-26

**Authors:** Harri Lähdesmäki, Alistair G. Rust, Ilya Shmulevich

**Affiliations:** Institute for Systems Biology, Seattle, Washington, United States of America; University College London, United Kingdom

## Abstract

An important problem in molecular biology is to build a complete understanding of transcriptional regulatory processes in the cell. We have developed a flexible, probabilistic framework to predict TF binding from multiple data sources that differs from the standard hypothesis testing (scanning) methods in several ways. Our probabilistic modeling framework estimates the probability of binding and, thus, naturally reflects our degree of belief in binding. Probabilistic modeling also allows for easy and systematic integration of our binding predictions into other probabilistic modeling methods, such as expression-based gene network inference. The method answers the question of whether the whole analyzed promoter has a binding site, but can also be extended to estimate the binding probability at each nucleotide position. Further, we introduce an extension to model combinatorial regulation by several TFs. Most importantly, the proposed methods can make principled probabilistic inference from multiple evidence sources, such as, multiple statistical models (motifs) of the TFs, evolutionary conservation, regulatory potential, CpG islands, nucleosome positioning, DNase hypersensitive sites, ChIP-chip binding segments and other (prior) sequence-based biological knowledge. We developed both a likelihood and a Bayesian method, where the latter is implemented with a Markov chain Monte Carlo algorithm. Results on a carefully constructed test set from the mouse genome demonstrate that principled data fusion can significantly improve the performance of TF binding prediction methods. We also applied the probabilistic modeling framework to all promoters in the mouse genome and the results indicate a sparse connectivity between transcriptional regulators and their target promoters. To facilitate analysis of other sequences and additional data, we have developed an on-line web tool, ProbTF, which implements our probabilistic TF binding prediction method using multiple data sources. Test data set, a web tool, source codes and supplementary data are available at: http://www.probtf.org.

## Introduction

Transcriptional regulation is a central control mechanism for many biological processes. Transcriptional regulation generally involves DNA-binding proteins, transcription factors (TFs), that control gene expression by binding to short regulatory sequence motifs in gene promoters [1]. DNA-binding specificities of TFs are encoded in their DNA-binding domains that specialize them to recognize and bind specific types of binding sites. This mechanism is the basis of control in complex transcriptional regulatory networks. Revealing these regulatory mechanisms is one of the key problems in understanding genome-wide transcriptional regulation. Although experimental studies and computational approaches are extending our knowledge of TF binding specificities, relatively little is known about genome-wide binding of TFs to gene promoters. Thus, TF binding prediction remains an important problem in computational biology.

Computational approaches to TF binding site analysis can be divided into two categories, *discovery* and *prediction*. Motif *discovery* focuses on searching for novel binding motifs from a collection of short sequences that are assumed to contain a common regulatory motif. Several algorithms have been proposed for motif discovery (for a recent review and comparison, see [2], [3]). Accurate motif discovery is difficult in general, but incorporating additional information to guide the search for novel sequence signals can improve performance. Such additional data sources include, among others, information about co-regulated genes [4], evolutionary conservation [5], [6], physical binding locations as measured by chromatin immunoprecipitation on chip (ChIP-chip) [7]–[9], information on the structural class of TFs [10], and nucleosome occupancies [11], [12].

TF binding *prediction*, in turn, makes use of given DNA-binding specificities to predict putative TF binding sites. The binding preferences can either be the output of a motif discovery algorithm or they can be experimentally measured, such as those reported in curated databases (TRANSFAC [13] and JASPAR [14]). Regardless of the data source, binding site prediction typically requires some information about binding specificities and is therefore dependent on previous analysis. Current knowledge of binding preferences already allows useful predictions to be made genome-wide. Moreover, several novel measurement techniques to measure DNA-binding specificities have recently been developed [15]–[19]. For example, Berger *et al.* [16] have developed a protein binding microarray (PBM) technology to measure binding preferences to all *k*-mers, *k* currently being 10 base pairs. These new techniques are rapidly expanding currently available databases by providing estimates of binding specificities of virtually any TF in a high-throughput manner. Consequently, they also offer an approach for rapid and sensitive identification of all TF binding sites genome-wide. In particular, high-throughput screening of TF binding specificities combined with accurate TF binding prediction provides a viable, condition independent, alternative to somewhat complex ChIP-chip experiments [20]. At the same time, however, there is a growing need for accurate TF binding prediction methods.

Although motif discovery methods are relatively well-developed, the TF binding prediction problem has attracted less attention. Most of the previous binding site prediction tools have been formulated as hypothesis testing methods, where a significance value of TF binding at a specific sequence position is obtained by comparing a test statistic to a null distribution [21]–[28], and possibly correcting the significance level for multiple testing. Traditional scanning methods for TF binding site prediction are known to perform relatively poorly in that they typically have an excessively high false positive rate (see [29]). This reported poor performance is not directly a shortcoming of previous prediction methods but has more to do with the fact that models to represent binding motifs and background sequences alone do not contain sufficient information for accurate binding site detection. This suggests that one possible approach to improve binding site prediction is to develop better motif (and background) models than the currently used position specific frequency model (PSFM) for binding sites and Markovian models for background. For example, observed dependencies between binding site nucleotides [30], [16] can be incorporated into motif models [31]. However, the use of more complex models, such as general Bayesian networks, is found to be challenging [32]. While developing better motif and background models is important, another more general direction aims at making use of several additional information sources, in a similar manner as has been done in the context of motif discovery (see above) to improve TF binding prediction.

Here, we formulate a probabilistic framework for TF binding prediction that differs from the standard hypothesis testing approaches in three important ways. First, the proposed framework is probabilistic in nature and thus outputs a probability of binding (as opposed to a *p*-value), which directly reflects our belief of gene's promoter having a binding site. We introduce both likelihood and Bayesian inference methods that naturally allow regularization via various prior distributions. Secondly, the proposed method answers the question of whether the whole promoter has a binding site, as opposed to reporting a *p*-value for every possible position in the sequence. But it is also straightforward to modify our methods to estimate TF binding separately for each base pair position as well, which we also consider. Since we process each promoter as a whole, in addition to assessing physical TF binding at the individual locations, our computational predictions provide insights into the functional role of a TF in the regulatory program of a target gene. The rationale for this is the fact that the higher binding probability anywhere on the promoter (not just in a particular location) implies higher probability of a regulatory relationship. Thirdly, and most importantly, we propose a principled way of combining multiple data sources, such as evolutionary conservation, regulatory potential, CpG islands, nucleosome positioning, DNase hypersensitive sites, ChIP-chip, and other prior knowledge, into a unified probabilistic framework. Moreover, the proposed data fusion framework is extremely versatile and thus, allows incorporating practically any additional (future) information sources that are indicative of TF binding sites at the genome level.

To validate our computational methods, we constructed a test set of annotated binding sites in mouse promoters from existing databases [13], [33]–[35]. We demonstrate that our probabilistic inference framework significantly improves TF binding predictions. We also test our probabilistic inference method by applying it to all known mouse promoters. These genome-wide results, which are made publicly available, indicate a sparse connectivity between transcriptional regulators and their target promoters. To provide easy access to this method, we have also implemented a web tool, ProbTF, which allows users to analyze their own promoter sequences and additional data sources.

Because our proposed computational framework is based on probabilistic modeling, it provides an intuitive interpretation (i.e., probabilities, not *p*-values). Our probabilistic formulation also provides regularization to the inference problem via several informative prior distributions, hence further improving performance. Results on a carefully constructed test set show that the proposed computational methods significantly improve performance when compared to previous binding site prediction methods. This is partly due to the fact that each promoter sequence is analyzed as a whole (i.e., TF binding is not assessed independently at each nucleotide position), taking advantage of multiple binding sites. The most important ingredient, however, is the principled incorporation of multiple additional data sources.

We construct our basic probabilistic framework using commonly used models for binding and non-binding sites, although generalizations to more complex models are straightforward. Consequently, our initial formulation is similar to other previously proposed TF binding prediction methods [36]–[38] and has perhaps even more in common with general motif discovery methods [6], [39]–[45]. Notably, the differences are that we further extend our basic TF binding prediction method into a Bayesian setting, incorporate multiple motif models, consider combinatorial regulation with multiple TFs, combine both forward and reverse strands into the modeling framework, provide a way to simultaneously estimate binding probabilities to the whole promoter and at single nucleotide resolution and, most importantly, provide a principled statistical integration of multiple data sources.

Although applications that combine binding site prediction with other data sources, especially “phylogenetic footprinting,” are plentiful (see e.g [46], [47], or [29], [48] for review), we are not aware of other probabilistic frameworks for TF binding modeling that can combine several additional data sources at the genome level. Related probabilistic data fusion approaches to TF binding prediction are introduced in [49], [50]. For example, the method of Beyer *et al.* [49] provides a general, higher-level, naive Bayes approach to integrate ChIP-chip data with several additional evidence sources. Our approach is different in that we integrate multiple data sources at the genome level, which is indeed necessary for incorporating nucleosome information, regulatory potentials, etc. We also note that our probabilistic predictions can be further used in other methods, such as the one of [49]. Other related previous methods include, among others, probabilistic methods for combining sequences and microarray data [51], [52], general frameworks for data integration (see [53]), and a supervised method for binding site prediction [54], [55]. The most closely related previous method is that by Thijs *et al.* [42], although that was originally introduced in the context of motif discovery and without an option for data fusion. Note that depending on what additional data sources are available, our method can be applied with either zero or any number of additional information sources.

Although our general aim is to integrate as many lines of evidence as possible into TF binding prediction, we restrict our focus to those data sources that contain useful information for TF binding at the genome level. For example, we do not consider functional data sources, such as gene expression or protein level measurements, or descriptive higher-level information, such as gene ontology. Despite the fact that gene or protein expression measurements alone can be informative of transcriptional regulatory relationships and, therefore, indirectly informative of TF binding as well, we will not include those data sources into our modeling framework. For example, proper modeling of gene expression or protein level measurements does inevitably require the use of a predictive network model, such as a (dynamic) Bayesian network [56]–[58], a system of ordinary differential equations or a stochastic kinetic model [59]–[61]. However, even more comprehensive modeling approaches that integrate all sequence level data sources with functional measurements and gene ontology information become much easier to tackle because our TF binding prediction are probabilistic. Probabilistic methods similar to those presented in [62]–[64] are practically straightforward to apply using our sequence level predictions as a building block (see ‘Discussion’ Section for more discussion). From a more general point of view, our approach has much in common with general strategies to infer transcriptional regulation from multiple data sources (see [62]–[65] for representative examples). We expect that the method described herein will also prove to be a useful building block in comprehensive transcriptional regulatory modeling.

## Results

### Probabilistic modeling for binding prediction

To motivate the results presented in this section, we briefly outline our computational methods—first for the basic probabilistic formulation without any additional data sources and then for general data fusion. A detailed description of the methods is presented in ‘Materials and Methods’ Section.

The most commonly used probabilistic models for binding sites and background sequences, on which our methods are also built, are the position specific frequency matrix (PSFM) model [66], [21] and the Markovian model [67], respectively. Our choice of using PSFM model for binding sites is arbitrary. The same modeling framework can be extended to virtually any binding site model. Although we focus on TF binding prediction, our computational formulation is perhaps more closely related to probabilistic motif discovery methods (see e.g [6], [39]–[45]). Note, however, that our goal is not *de novo* motif discovery but probabilistic inference of TF binding, given some *a priori* information about TF binding specificities and background sequence properties. Because we assume to have prior knowledge of TF specificities we can use a different approach in our computation (compared to motif discovery methods) to better address our goal of estimating TF binding. Further, these standard motif and background models are combined in a probabilistic framework with multiple additional data sources that are indicative of TF binding or transcriptional regulation in general.

**Figure 1:**
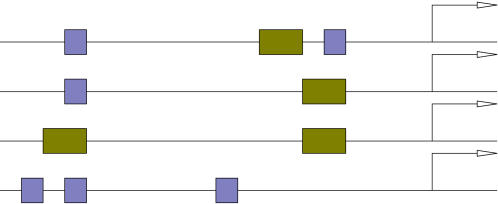
An illustration of four different binding site configurations for a TF that is associated with two motif models (blue and green boxes). The diagram illustrates the upstream promoter region for a gene, where the direction of transcription is indicated by the direction of the arrows. The arrows are located at the transcription start sites.

Motif and background models are denoted by θ and φ, respectively. For the cases where a TF is associated with more than one motif model, we denote multiple motifs by Θ = (θ^(1)^,…,θ^(*m*)^). A key (unknown) quantity is the number of binding sites *Q* in a promoter sequence *S* = (*s*_1_,…,*s_N_*). Instead of fixing *Q* = *c* binding sites to particular positions, we consider (sum over) all possible non-overlapping motif start positions *A* = {*a*_1_,…,*a_c_*} and configurations π∈{1,…,*m*}*^c^* and weight different combinations according to their probability. This is illustrated in Figure 1, which shows four different combinations of the number of binding sites *Q*, positions *A* and configurations π for a TF that is associated with two motif models (blue and green boxes).

Given *S*, Θ and φ, we are interested in computing the probability of having *c* = 0,1,… binding sites
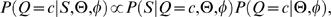
where the term *P*(*S*|*Q* = *c*,Θ,φ) involves summing over all motif positions *A* and configurations π for *c* binding sites. The prior *P*(*Q* = *c*|Θ,φ) reflects our prior belief of having *c* binding sites. The probability of binding can be assessed by the probability of having at least one (or any other higher number, if so decided) binding site *P*(*Q*>0|*S*,Θ,φ). A computationally efficient recursive algorithm to compute *P*(*Q*>0|*S*,Θ,φ) is described in ‘Materials and Methods’ Section. Our basic formulation can be viewed as a direct generalization of that by Thijs *et al.* [42] who proposed a practically equivalent framework for a single motif model. Similar probabilistic modeling frameworks have also been constructed using hidden Markov models [36]–[38], [44].

Because motif models are typically constructed from a relatively small number of experimentally verified binding sites (or from an output of a motif discovery algorithm) they can contain a considerable amount of uncertainty. Thus, it is also useful to consider a Bayesian approach where Θ and φ are random variables. Given *S*, *A* and π and applying Bayes' rule gives
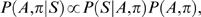
where *P*(*S*|*A*,π) is the marginal likelihood obtained by integrating over parameters Θ and φ. The probability of binding can again be assessed by the probability of having at least one binding site, i.e., 1−*P*(*A* = Ø,π = Ø|*S*). For the Bayesian approach we develop a Markov chain Monte Carlo (MCMC) estimation method (see ‘MCMC estimation for Bayesian inference’ Section for details). Another important benefit of using a Bayesian approach is that with the proposed MCMC sampling strategy one can solve more complex inference problems (and with more complex prior distributions) than with the efficient recursive algorithm for the likelihood method. In particular, MCMC sampling also makes it possible to jointly estimate binding probabilities to the whole promoter and individual base pair locations.

### Performance on real sequences

We demonstrate our computational methods on a carefully constructed test set that contains annotated binding sites in 47 mouse promoters. For the positive cases we use those TF-promoter pairs that contain an annotated binding site for a TF in a promoter. Negative cases consist of TF-promoter pairs that (are expected to) have no functional binding sites. Although negative sets in general are likely to contain some true binding sites (those that are not yet discovered), that does not invalidate our performance evaluation but merely introduces a degrading bias in our results. Similarly, some of the annotated binding sites may have been used to construct the PSFM models that we use in our analysis and that in turn can introduce an optimistic bias into our results. The above-mentioned biases are impossible to avoid in practice. Fortunately, this is not a major issue, especially as long as we use the same test set and the same PSFM and Markovian background models for all the new and previously published methods that we compare. The full details of the test set can be found in ‘Data’ Section. For the binding specificities, we use (scaled) motif models from TRANSFAC Professional version 10.3. We measure performance using standard receiver operating characteristics (ROC) curves that plot the fraction of true positives (sensitivity) versus the fraction of false positives (complementary specificity), see e.g [68]. We also use the area under the curve (AUC) measure that summarizes and represents the ROC with a single number. Given the probabilistic nature of our method, we also found it instructive to visualize the distribution of estimated binding probabilities for positive and negative test cases.

### Comparison of background model orders

**Figure 2:**
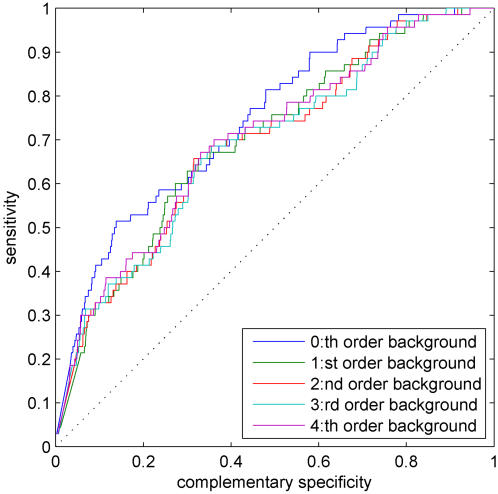
The standard ROC curves for the basic likelihood-based method with varying Markovian background model orders, *d*∈{0,1,…,4}.

Before proceeding to more interesting results, we first test the effect of some of the parameters in our probabilistic formulation. A natural parameter to start with is the order of the Markovian background model. Several authors have reported that the use of higher-order background models improves motif discovery [67], [69]. Figure 2 shows ROC curves for our test set using the basic likelihood-based probabilistic method with varying Markovian background model orders, *d*∈{0,1,…,4}. Figure 2 shows somewhat surprisingly that, overall, TF binding prediction does not seem to depend greatly on the background model order. The best overall performance, according to AUC, is achieved with *d* = 0. For consistency, we use *d* = 0 in all our simulations.

Another useful preliminary test is to vary the prior probability of having *Q* = 0 binding sites as well as the parameter κ, which specifies how fast the prior probability of having *Q*>0 binding sites approaches zero (see ‘Materials and Methods’ Section for more details). In general, these parameters only affect the overall bias of the posterior binding probabilities: larger values of *P*(*Q* = 0|Θ,φ) and smaller values of κ bias probabilities towards small values, and vice versa (results not shown).

### Comparison of likelihood and Bayesian approaches

**Figure 3:**
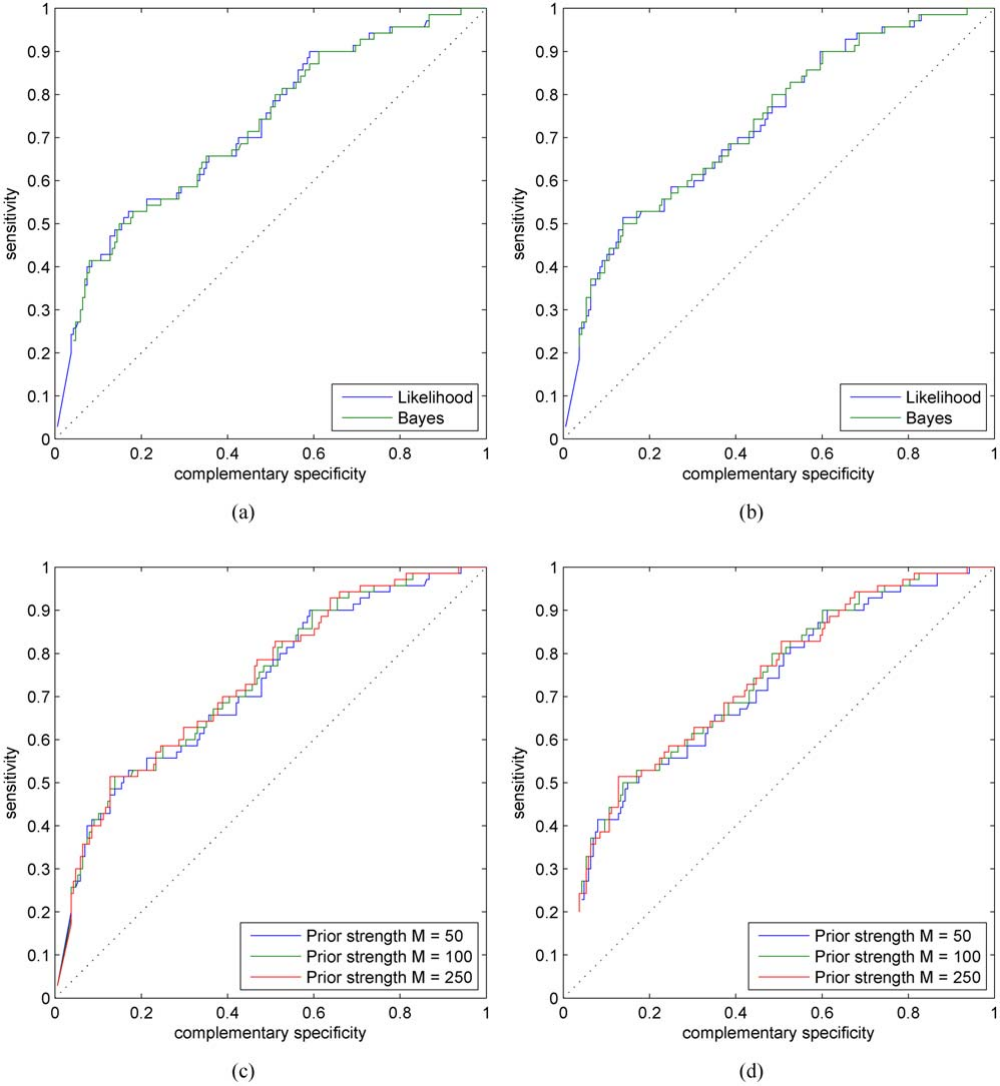
ROC curves for the likelihood and Bayesian probabilistic methods with varying prior strengths. (a) *M* = 50. (b) *M* = 100. Results for (c) likelihood-based and (d) Bayesian methods for various values of *M*.

Adopting a Bayesian approach allows the modeling of uncertainty in the model parameters as well. Although we use the Dirichlet prior distribution for both the motif and background model parameters, we are primarily concerned with the uncertainty in the motif models, since the background model parameters are estimated from a much larger data set. Hyperparameters of the Dirichlet prior control the amount of uncertainty in the motif model parameters and that can also provide regularization for the inference problem (see [70] for a discussion in the context of Bayesian networks). For consistency, we use the same hyperparameters to obtain the scaled PSFMs for the likelihood-based approach. Figure 3 shows the ROC results for the likelihood and Bayesian methods with varying prior strengths (see ‘Materials and Methods’ Section for more details). In the basic simulations setting without any additional data sources, the likelihood and Bayesian methods perform almost identically.

**Figure 4:**
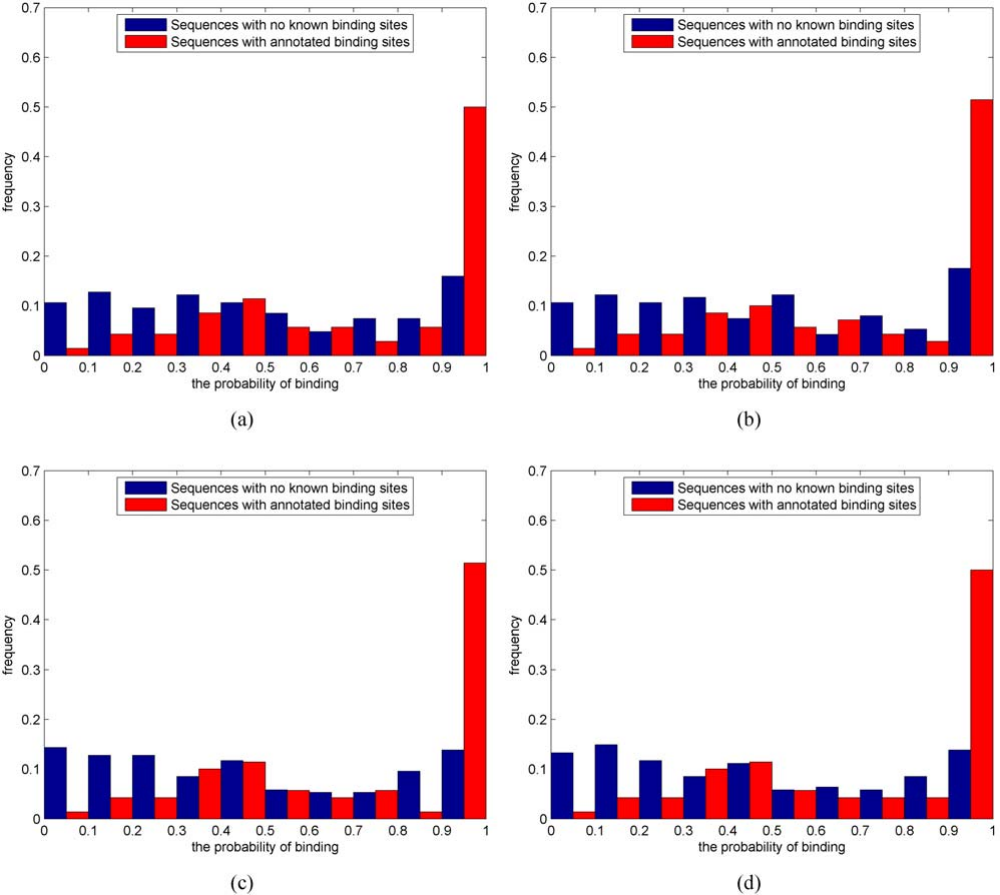
Histograms of the estimated binding probabilities for the likelihood and Bayesian methods with varying prior strengths. (a) Likelihood *M* = 50. (b) Bayesian *M* = 50. (c) Likelihood *M* = 100. (d) Bayesian *M* = 100. *x*-axes correspond to the estimated binding probability and *y*-axes show the fraction of negative (blue) and positive (red) test cases. Histogram bin edges are located at 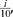, *i* = 0,1,…,10, although the two histograms are shown side by side.

Figure 4 shows histograms of the estimated binding probabilities for the likelihood and Bayesian methods for prior strengths *M* = 50 and *M* = 100. A general character of the histograms is that the binding probabilities of annotated sites (red bars) are biased towards high values whereas the binding probabilities of negative cases (blue bars) are approximately uniformly distributed. Smaller prior strengths correspond to more uncertain motif models and that, in turn, allows the data (i.e., promoter sequence) to have a stronger effect on the posterior motif model. Typically, smaller prior strengths also bias overall posterior probabilities towards higher values, especially for the negative set (compare blue bars in Figures 4 (a–b) with the ones in (c–d)). In terms of ROC curves and AUC measures, proposed methods are insensitive to small deviations in prior strengths (within a range of reasonable values). Prior strength *M* = 100 seems to provide good results and this value was used in all our simulations for consistency.

### Comparison with traditional promoter scanning

**Figure 5:**
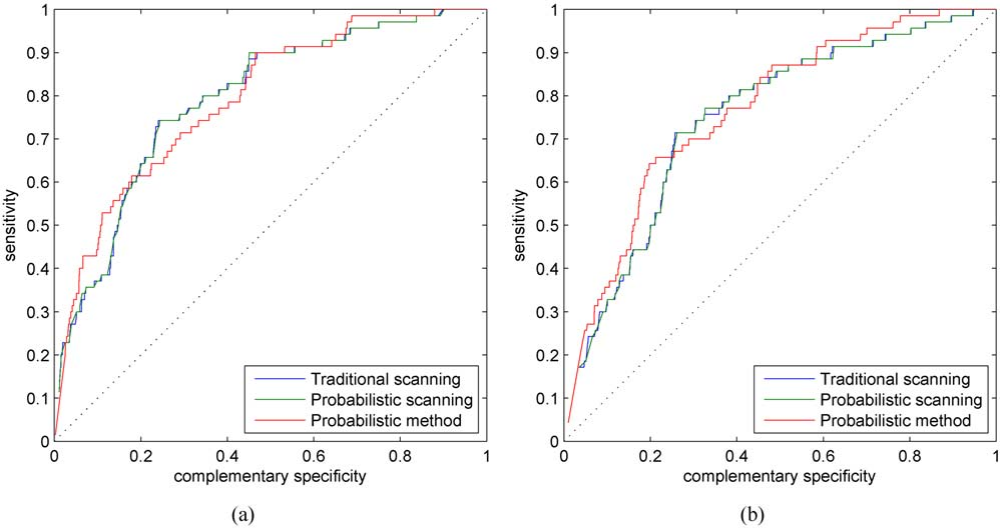
ROC curves for the likelihood-based probabilistic method (red), traditional scanning (blue), and a probabilistic scanning-based method that outputs a probability of binding (green). The background model order is (a) *d* = 0 and (b) *d* = 1.

In order to better assess the performance of the proposed methods, we also compare our basic likelihood method with traditional promoter scanning (see [21]–[24]) and a probabilistic scanning-based method that assesses the probability of binding [63] (see ‘Comparison with other methods’ Section for more details). Due to a fundamental problem of hypothesis testing approaches, they are prone to systematically finding more significant binding sites in longer promoter sequences just by chance. This inherent bias could be accounted for to an extent by correcting for the multiple testing issue. That would be remarkably challenging because all significance values corresponding to a PSFM model should be corrected simultaneously and that would easily result in millions of *p*-values per PSFM model. An even more severe issue, however, stems from the fact that exact promoter regions are unknown practically for all the genes. Thus, a particular choice of the promoter regions, especially their lengths, has a potential to impose a significant biasing effect. The easiest and safest way to guarantee a fair and unbiased comparison between different methods is to shorten all the promoter sequences in our controlled test set to have approximately the same length (but we also compare different methods without changing the promoter lengths for comparison purposes). Figure 5 shows ROC results for the three methods with two different background model orders. Overall, the performance of all three methods is similar, which is to be expected since they are based on the same motif and background models. However, our probabilistic method performs better for small values of false positives (about <0.2). This is partly due to the fact that our method makes inference using multiple binding sites together and hence, it is able to assign higher probability to sequences that have multiple annotated binding sites. Performance differences become more significant when we integrate multiple data sources into TF binding predictions in the next section. In traditional hypothesis testing based approaches, one has typically been interested in a region of the ROC curves that corresponds to a remarkably small false positive rate, say <0.01. This is mainly due to the fact that each PSFM and nucleotide location pair is assigned its own significance value and therefore even a tiny false positive rate makes the results look like each factor binds to practically every promoter. Fortunately, this issue becomes less severe in our modeling framework since each promoter is processed as a whole (i.e., a single binding probability per TF-promoter pair), although later on we also estimate binding probabilities at a single nucleotide resolution. Further, and perhaps more importantly, one of our main goals is full probabilistic modeling, where different evidence sources are combined in a seemingly continuous fashion (i.e., without making calls of ‘binding’ or ‘not binding’ at each step of the inference process). For example, practically all parts of the ROC curve become equally important when our sequence-based probabilistic modeling is combined with gene expression data and other information sources. Supplemental Figure S1 shows the same comparison results as in Figure 5, but without forcing the promoter lengths to be equal. These results show that when promoters have varying lengths, our probabilistic method that processes each promoter as a whole performs even better when compared to scanning-based approaches.

### Probabilistic integration of multiple data sources

Many TF binding sequences are relatively short and non-unique and hence, the expected number of their occurrences in a genome by chance is high. These presumably non-functional binding sites cause traditional TF binding site prediction methods to have unacceptably high false positive rates. Although the above probabilistic formulation provides a principled framework for TF binding inference and allows regularization via a Bayesian approach, being built on the same modeling framework as other methods (PSFM motif and Markovian background models), it is also vulnerable to identifying non-functional sites.

A natural way to improve specificity of TF binding site predictions is to make use of additional biological information in the inference. This idea has been proposed in several articles where, for example, promoter scanning with PSFMs has been constrained to only those parts of the genome that are highly conserved, i.e., conservation scores exceed a threshold (see reviews in [29], [48]). However, not all binding sites are conserved [29]. Therefore, using additional data in such a binary fashion, is likely to miss some binding sites and does not provide the most efficient use of the data. A key advantage of our formulation is that it provides a principled, probabilistic framework for incorporating multiple data sources.

A number of additional information sources can be useful for predicting TF binding. First, functional binding sites are typically evolutionarily conserved [71], albeit with the caveat described in the previous paragraph. Alignments of interspecies genomes combined with other modeling efforts can be used to assess the probability that a certain genomic location is under evolutionary selection, and thus more likely to be a functional binding site. We use PhastCons [72] to assess the probability of conservation. PhastCons uses genome alignments of 17 species and a continuous-time Markov model for nucleotide substitutions and a two state phylo-HMM model to compute posterior conservation probabilities.

Second, in addition to evolutionary conservation, methods exist to assess whether a conserved sequence is neutral or functional. This more detailed information, often called regulatory potential, has a potential to distinguish neutral sequence regions from the functional ones, even within conserved parts of sequences. Regulatory potential scores (log-likelihoods) are obtained using ESPERR [73] that also makes use of multiple genome alignments. After appropriate dimension reduction and alphabet selection, ESPERR applies two variable order Markov models to estimate likelihoods of regulatory and neutral sites. The two Markov models are trained from a set of known regulatory and neutral sites which makes ESPERR essentially a discriminatory method.

Third, while evolutionary conservation can help in discriminating functional binding sites that are more prevalently located on conserved parts of the genome from presumably non-functional sites on non-conserved regions, it does not explain the mechanism by which a TF is guided to its functional site. A hypothesis is that this process is controlled by the intrinsic nucleosome organization of genomes [74], [75]. The likelihood of binding to a non-functional binding site can be decreased by locating a stable nucleosome over those genomic regions while keeping functional sites accessible for TFs, i.e., free of (stable) nucleosomes. The nucleosome occupancy probabilities can be computed using a method by Segal *et al.* [74], which uses a Markov model whose parameters are estimated from a set of known nucleosome locations.

Although we primarily focus on the three additional evidence sources mentioned above, other information sources can also be directly included into our modeling framework. For example, a general sequence feature of many promoters, and thereby a feature of binding sites within promoters as well, is that they typically have a high CpG dinucleotide content [75]. Binding sites are also commonly found to be organized into clusters (for a review, see [76]). Furthermore, other more important, direct evidence sources include experimental measures of TF binding as measured by chromatin immunoprecipitation on chip [77], [9] and DNase hypersensitive sites [78].

From a computational point of view, we assume that each additional data source is in the form *D* = (*P*(1),…,*P*(*N*)), where *P*(*i*) denotes the probability that the *i*th nucleotide has one of the above sources of evidence. Although not all data sources are in the form of probabilities they can often be interpreted or transformed such that they conform to this format. Additional data *D* is assumed to be conditionally independent of the sequence *S* (given *A*, π, Θ and φ) and independent of the motif and background models, i.e.,
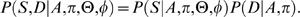
(1)


The intuitive rationale for defining the term that captures the additional data, *P*(*D*|*A*,π), is to assign higher probabilities for those configurations (*A*,π) that are located in regions that are more likely (in light of additional data *D*) to contain functional binding sites. A similar data fusion technique works in a Bayesian framework as well
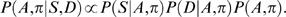
(2)


We extend the above framework to multiple data sources *D_i_*, 1≤*i*≤*N_D_*, by combining them using a standard weighting scheme prior to applying Equations (1–2). The details of the computational methods for incorporating multiple data sources are described in ‘Combining multiple information sources’ Section.

### Performance on real multiple data sources

**Figure 6:**
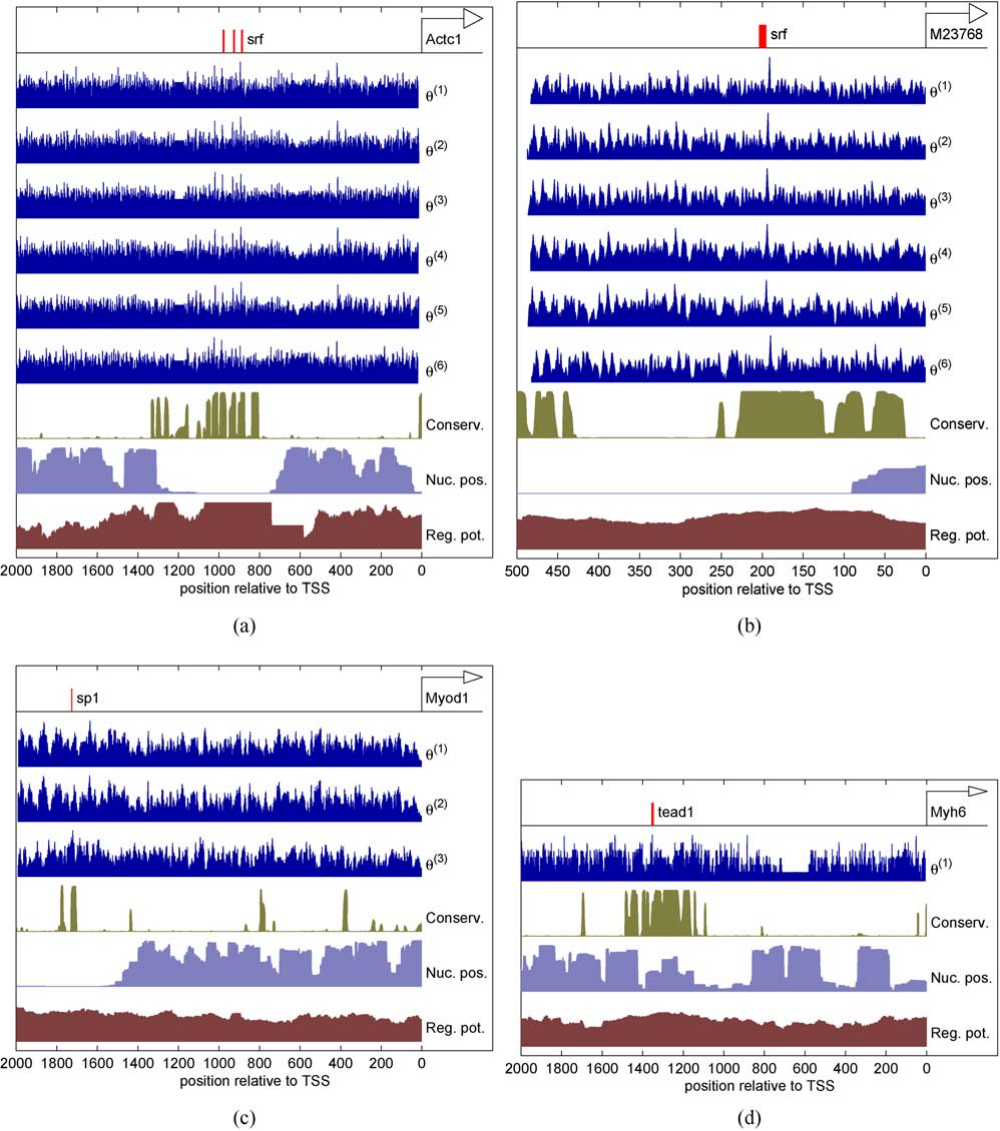
An illustration of the data fusion for TF binding prediction. (a) Annotated binding sites for SRF on Actc1 promoter. (b) Annotated binding site for SRF on M23768 promoter. (c) Annotated binding site for SP1 on Myod1 promoter. (d) Annotated binding site for TEAD1 on Myh6 promoter. Figure keys are as follows. θ^(*i*)^: motif models for each TF, Conserv.: sequence conservation probabilities computed by PhastCons [72], Nuc. pos.: nucleosome occupancy probabilities estimated by a yeast nucleosome model from [74], and Reg. pot.: regulatory potential log-likelihood scores from [73]. The additional evidences range between 0 and 1. Promoters sequence lengths are 2000 base pairs in (a), (c) and (d), and 500 base pairs in (b). See text for more details.

The data fusion problem is illustrated in Figure 6 for four TF-promoter pairs. The first row in each subplot shows the annotated binding site(s). For illustration purposes, the next rows show the log-likelihood scores of motif model(s) θ^(*i*)^ to the background model φ, 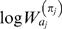 (see Equation (11) for details). The last three rows show the probabilities of conservation, nucleosome occupancy and the regulatory potential. Figure 6 illustrates some general characteristics of the data. First, the highest log-likelihood score 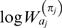 is not always obtained at the annotated site but on other, possibly non-functional, positions. Second, for some TFs, such as SRF, motif models θ^(*i*)^ are highly correlated whereas for other TFs, such as SP1, motif models produce “scores” which are distinct from each other. Finally, many of the annotated sites are also associated with a high probability of conservation and regulatory potential and with a low probability of nucleosome occupancy. This correlation is not expected to be perfect though since only about 50% of the functional binding sites are assessed to be conserved (see [29]). Our goal here is to make principled probabilistic inference from these numbers and to output a single probability of binding for the promoter as a whole (i.e., transcriptional regulation). We will also compute the probability of having a binding site at each nucleotide position later on.

### Comparisons between different additional data sources

**Figure 7:**
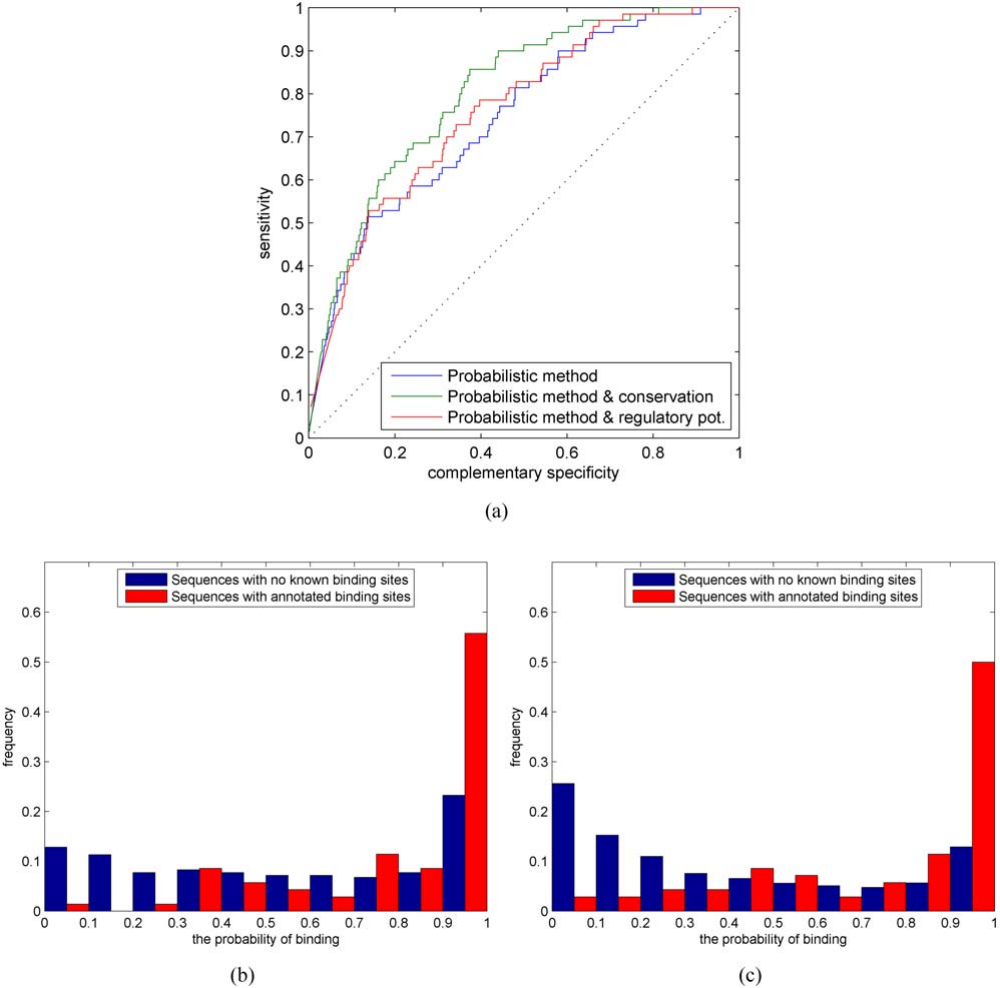
(a) ROC curves for the likelihood-based method (blue) when combined with a single additional information source: regulatory potential (red), and evolutionary conservation (green). Histograms of the estimated binding probabilities for the likelihood-based method when combined with (b) regulatory potential and (c) evolutionary conservation.

Figure 7 (a) shows ROC curves for the likelihood-based method (blue) when combined with a single additional information source. The use of regulatory potential scores (red) already gives a marginal improvement for the TF binding prediction, but evolutionary conservation (green) significantly improves the detection performance. Although nucleosome occupancy probabilities seem to be low at annotated sites in Figure 6, static predictions of nucleosome positions are not sufficiently informative to help binding prediction. Perhaps the main difficulty in using the predicted nucleosome locations is that the nucleosome model is constructed for yeast (only models for yeast, chicken and human are available in [74]). However, as Narlikar *et al.* [11] have demonstrated in the case of motif discovery in yeast, nucleosome information can be informative, especially if combined with ChIP-chip data in a discriminatory setting. We also found out that information of CpG-islands does not improve binding predictions.

Figures 7 (b) and (c) show the corresponding histograms of the estimated binding probabilities. Combining the probabilistic method with evolutionary conservation produces the most discriminatory histogram (Figure 7 (c)) whereas regulatory potential data (Figure 7 (b)) assign higher probabilities to some of the negative case. This can also indicate that the negative sequences may indeed contain unannotated binding sites.

In these simulations, the scaling parameter for each additional data source (see ‘Combining multiple information sources’ Section for details) is chosen using a grid search over values *d*∈{0, 0.01,…, 0.5} and taking the one that produces the best AUC measure. We found *d* = 0.04 for conservation and *d* = 0.05 for the regulatory potential data, but the results are not sensitive to small deviations in the values of the scaling parameters. To verify that choosing scaling parameters by maximizing the AUC measure does not introduce an optimistic bias, we repeated the same simulation using stratified cross-validation. For conservation scores, the results remained virtually the same and for the regulatory potential the results are also similar (see supplemental Figure S2). ESPERR [73] is trained on a set of human genes, which is further expanded by mapping the set of human genes to orthologous mouse genes, amongst others. This extended data set partly overlaps with our test set. We verified that this overlap does not introduce any bias in our results by removing the overlapping genes from our test set and repeating the simulation (see supplemental material Text S1 and Figure S4).

**Figure 8:**
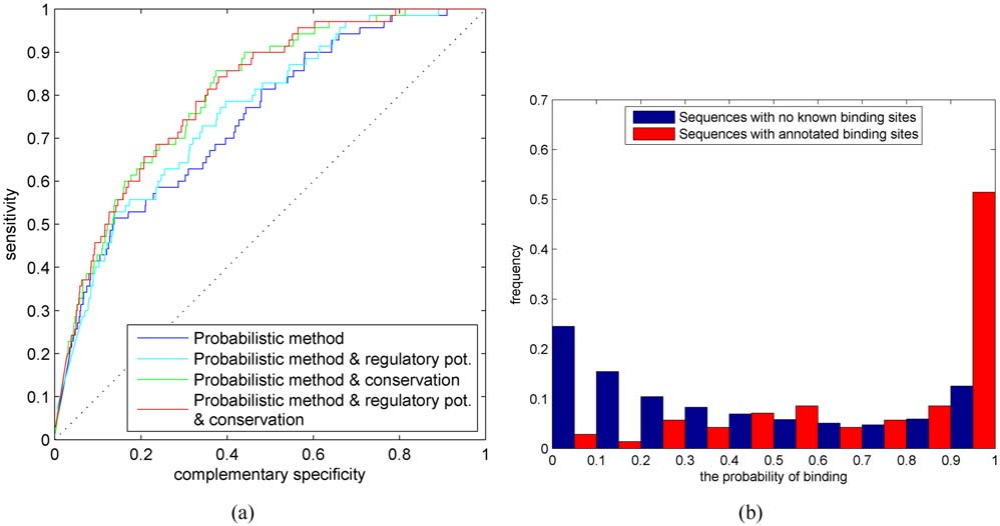
(a) ROC curve for the likelihood-based method (blue) when combined with evolutionary conservation (green), regulatory potential (cyan), and a combination of evolutionary conservation and regulatory potential (red). (b) Histogram of the estimated binding probabilities for a combination of conservation and regulatory potential.

Given the above promising results with a single additional data source, a natural question then is to study whether combinations of additional data sources further improve TF binding prediction. We consider the combination of conservation and regulatory potential for which the ROC curve as well as the corresponding histogram of the estimated binding probabilities are shown in Figure 8. Combining conservation and regulatory potential gives a minor improvement relative to using these data sources alone.

We used the same scaling parameters as above and tried a set of different weighting schemes and again chose the weighting parameter that gives the best AUC measure over grid *w*_2_∈{0.5, 0.52,…, 1}. We found *w*_1_ = 0.14 and *w*_2_ = 1−*w*_1_ = 0.86 for regulatory potential and conservation, respectively (see ‘Combining multiple information sources’ Section for details). Similarly to scaling, weighting is not sensitive to small deviations in the values of the weighting parameters. Weighting some of the data sources more heavily just biases the results towards those obtained using the particular single data source alone.

**Figure 9:**
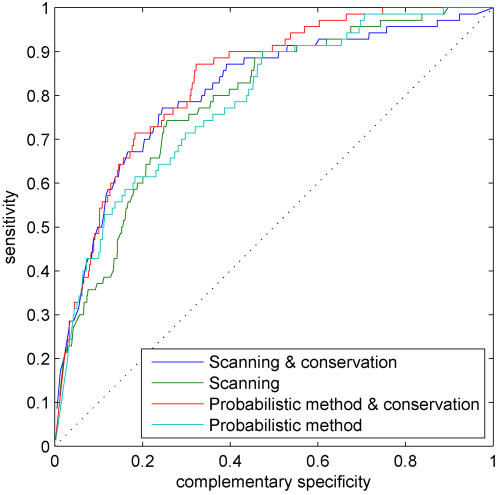
ROC curves for the traditional scanning (green), traditional scanning combined with thresholded conservation information (blue), probabilistic method combined with conservation information (red), and probabilistic method (cyan).

The standard practice has been to constrain the scanning with PSFMs to only those regions of the genome whose conservation probability (or score) is sufficiently high [29]. For comparison purposes, we applied the same strategy to our test set using a similar grid search for the optimal threshold as above. Figure 9 shows ROC curves for the traditional scanning with and without conservation data as well as our probabilistic method when combined with conservation information. These results demonstrate the fact that thresholding-based methods cannot achieve optimal performance since not all functional binding sites are conserved. This issue becomes more prevalent when more than one data source is integrated. Supplemental Figure S3 shows the same results as in Figure 9 but without forcing the promoter sequence lengths to be equal, in which case, our probabilistic method performs again even better than the traditional scanning. These results again demonstrate the potential bias of hypothesis testing based approaches.

Evolutionary conservation and regulatory potential are the most informative additional data source in our simulations, whereas estimated nucleosome locations or CpG-islands do not improve TF binding predictions. As mentioned above, the particular estimated nucleosome data that we use might not be optimal for our mouse test set. Once a mouse nucleosome model or high-throughput nucleosome data become available, they can be used in the same framework as well, likely improving TF binding predictions. For example, Narlikar *et al.* [12] have found that even low resolution measurements of nucleosome locations give a marginal improvement for motif discovery methods in yeast. Similarly, once more abundant ChIP-chip data becomes available, it can be incorporated into our modeling framework as well. This data fusion step can be performed in two ways: either use an approach where binding probabilities are estimated separately from ChIP-chip and sequence data and then combined (see [49]), or incorporate high-resolution ChIP-chip binding data (see [9]) directly into our model.

### Modeling combinatorial regulation

Gene regulation in higher organisms commonly requires multiple TFs. Thus, combinatorial regulation by several TFs is another important problem to study. The main difference between a single TF and multiple TFs regulating a gene is that combinatorial regulation requires all TFs to have at least one binding site for (at least) one of their motif models. Although multiple regulatory proteins can also form a complex and the complex can regulate a target gene via a single binding site, we only consider regulation via multiple binding sites, the single binding site case being similar with our previous analysis. Statistical inference for combinatorial regulation can be naturally addressed in our probabilistic framework. For that purpose, we propose to use both the likelihood and Bayesian methods (see ‘Combinatorial regulation’ Section for more computational details).

Combinatorial regulation by multiple TFs is less well-known and fewer combinatorial annotated binding sites are reported in databases or even in the literature (see [79]). We construct a sufficiently large test set for computational simulations from our test set of annotated binding sites. For each promoter sequence, we pair different TFs that have annotated binding sites on it and consider these TF pairs to jointly regulate a given gene. We add a further constraint that, for each pair of TFs, at least one annotated binding site pair is within a cluster, i.e., sufficiently close to each other, as is typically the case in real promoters as well [76]. We construct a negative set such that approximately half of the TF pairs have no annotated binding sites and half of the TF pairs have an annotated binding site for one TF but not for the other.

**Figure 10:**
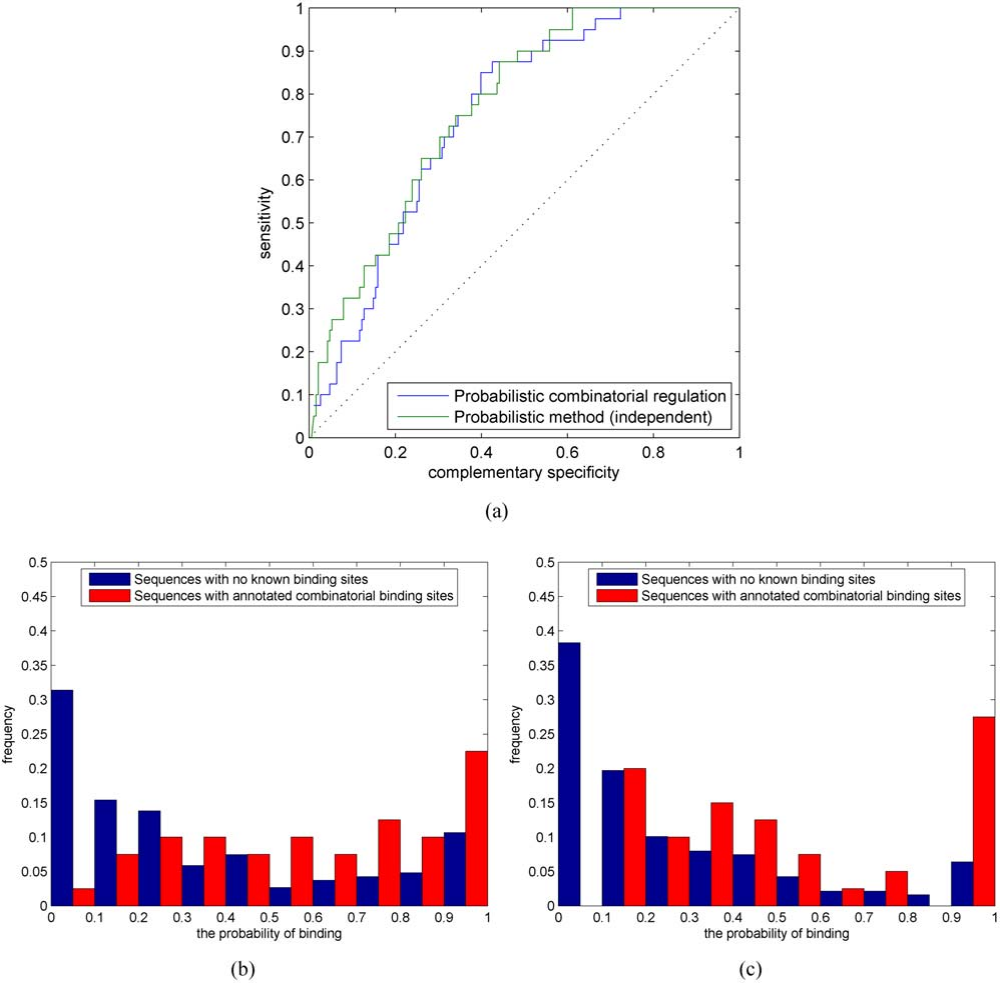
(a) ROC curves for combinatorial regulation using the Bayesian method (blue) and a naive likelihood approximation (green). Histogram of combinatorial regulation probabilities for (b) the Bayesian method and (c) naive likelihood approximation.

The problem of inferring combinatorial regulation among many TFs becomes computationally expensive because there are 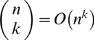 ways to choose *k* TFs from the set of *n* TFs. Inference for combinatorial regulation can only be done exactly using an MCMC sampler. This can become a problem since Bayesian inference is considerably slower than the likelihood-based inference. Therefore, in addition to Bayesian inference for combinatorial regulation, we also consider here a naive approximation that estimates the probability of combinatorial regulation by the product of individual TF binding probabilities. Figure 10 shows ROC results and the corresponding histograms for the two different methods. Surprisingly, the approximative method performs slightly better than the Bayesian method. Figures 10 (b) and (c) seem to suggest that the Bayesian method assigns higher probabilities to some of the negative cases and, thereby, results in slightly worse ROC curve than the likelihood method. Note that, given the more difficult problem of finding multiple weak sequence signals, histograms of combinatorial regulation probabilities for the positive set (red bars) now span the whole range from 0 to 1.

**Figure 11:**
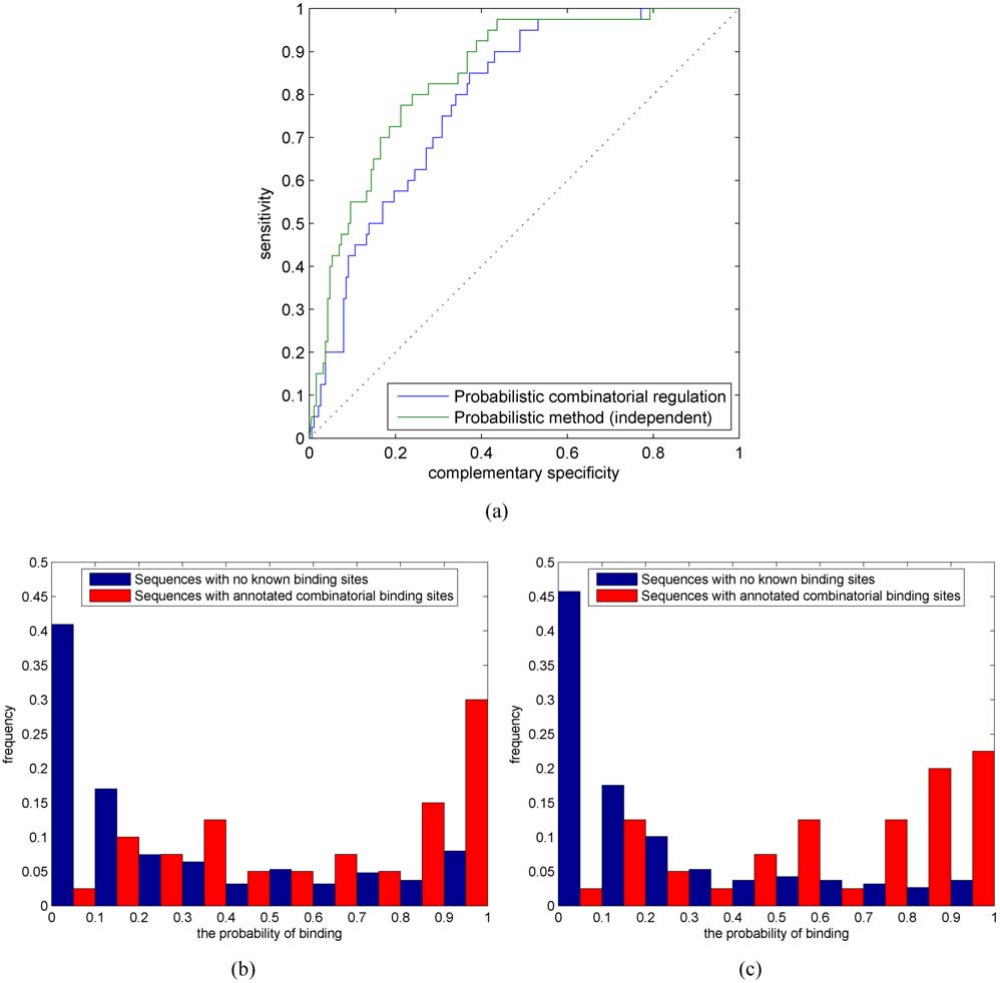
(a) ROC curves for combinatorial regulation using the Bayesian method with evolutionary conservation (blue) and a naive likelihood approximation with evolutionary conservation (green). Histogram of combinatorial regulation probabilities for (b) the Bayesian method with evolutionary conservation and (c) a naive likelihood approximation with evolutionary conservation.

As suggested by previous simulations, the detection of combinatorial regulation can be improved by incorporating additional data sources. We consider using evolutionary conservation for which the results are shown in Figure 11. Comparison of Figures 10 and 11 shows that additional data improves performance significantly. As in the case of no additional information, the naive likelihood approximation performs better than the Bayesian alternative. These results suggest that our proposed methods are well suited for inferring combinatorial regulation as well. Further, the naive likelihood approximation provides a computationally efficient alternative.

### Extensions to double-stranded DNA and single nucleotide resolution Single vs. both strands

**Figure 12:**
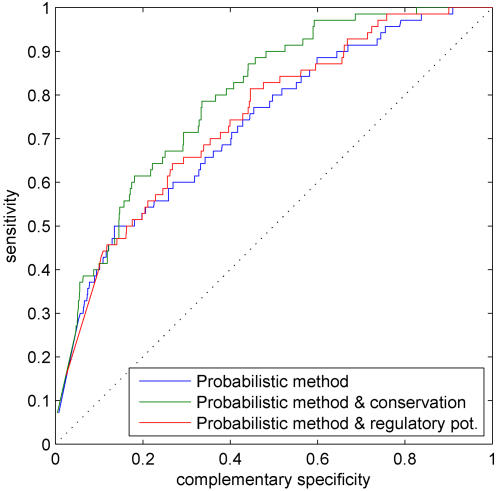
ROC curves for the likelihood-based method (blue) when both strands of the DNA are used and a single additional information sources is available: regulatory potential (red) and evolutionary conservation (green).

So far we have assumed that the direction of transcription is known and we have focused on analyzing only a single strand of the DNA. This is not always the case and, therefore, it is useful to generalize TF binding prediction methods such that they use both strands of the DNA. Our probabilistic methods generalize naturally to handle double-stranded DNA. This can be achieved simply by applying the aforementioned methods to both strands, either independently or simultaneously (computational details are described in ‘Single vs. both strands’ Section). To demonstrate performance of our methods on double-stranded DNA, we re-compute the results shown in Figure 7. Here we use a variant of the likelihood method that processes different strands independently. Figure 12 shows the resulting ROC curves. Results in Figures 7 and 12 are virtually identical which suggests that our proposed methods perform equally well on both single and double-stranded DNA.

### Predicting binding site positions

**Figure 13:**
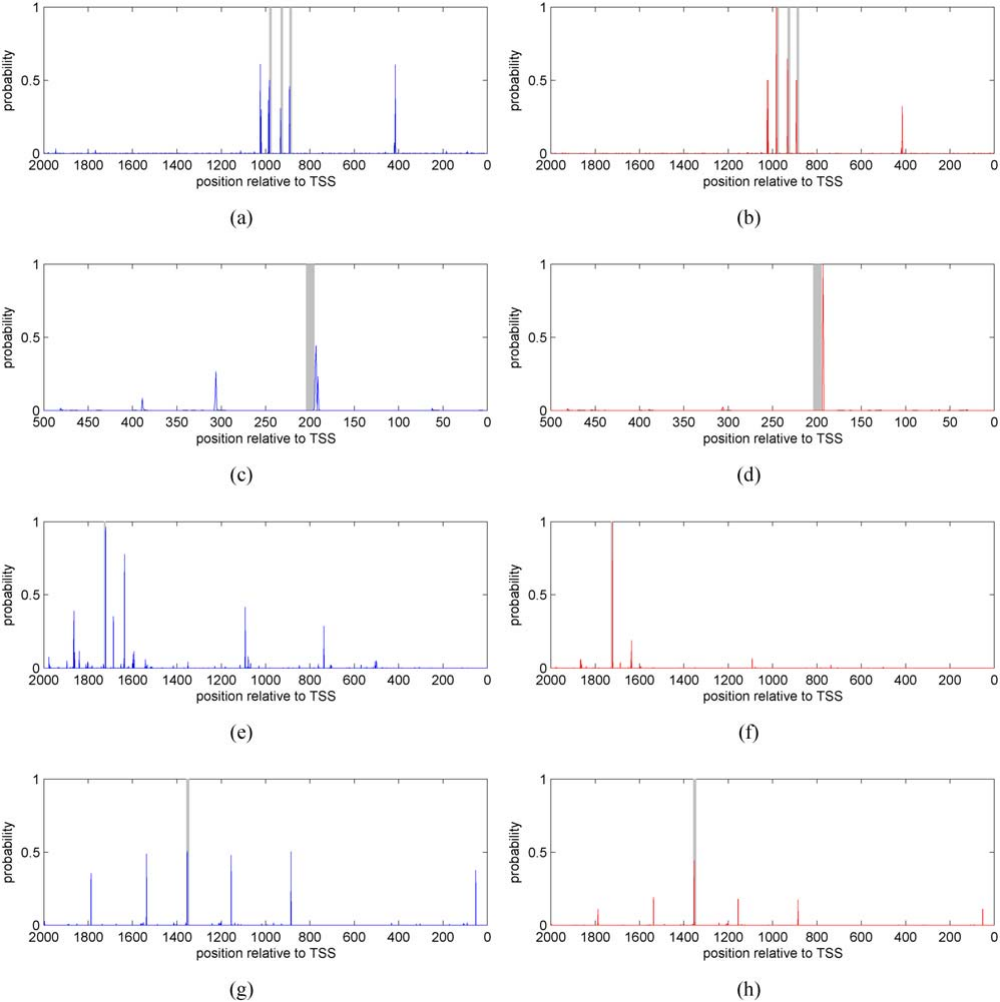
Estimated binding probabilities on a single base pair resolution for SRF on (a) the Actc1 and (c) M23768 promoters, (e) SP1 on the Myod1 promoter, and (g) TEAD1 on the Myh6 promoter without any additional information. Subplots (b), (d), (f) and (h) show the same results but with evolutionary conservation as the additional data source. The blue and red graphs indicate the start of the binding sites. The annotated binding sites are shown with gray vertical bars. These results correspond to Figure 6.

Our final simulation concerns inferring the probability of having a binding site at a single nucleotide position. Although our proposed methods are primarily designed to process each promoter sequence as a whole, it is also useful to be able to infer binding probabilities at a higher resolution, in particular, at each nucleotide position. Inferring the binding probabilities at each base pair location is more challenging from the computational point of view. In particular, the efficient recursive algorithm developed for the likelihood method cannot be applied. The inference can, however, be easily performed in our Bayesian framework using our MCMC sampler. The binding probability at each base pair location is achieved by integrating out all other locations (see ‘Binding probabilities at single nucleotide resolution’ Section more details). Figure 13 shows a representative result of binding probabilities for SRF on the Actc1 and M23768 promoters, SP1 on the Myod1 promoter, and TEAD1 on the Myh6 promoter, with and without evolutionary conservation as an additional data source. These results correspond to the data shown in Figure 6. The Bayesian inference (left column) is able to find and assign a high probability to all the annotated sites, although some presumably non-functional (i.e., non-annotated) sites are identified as well. The use of evolutionary conservation improves the performance of the Bayesian inference by “biasing” the binding sites towards conserved regions. In particular, all the annotated (resp. non-annotated) binding sites are assigned a higher (resp. lower) binding probability than without conservation data. It is also worth noting that our probabilistic inference method does not assign a zero probability to any of the possible binding sites. This is useful because the motif models are known to contain uncertainty and particularly because the binding prediction is based on noisy data. Hence, a probabilistic approach is more flexible and principled.

### Genome-wide Analysis

Encouraged by the above performance evaluations, we applied the proposed likelihood-based binding prediction method to the 2K base pair upstream promoter regions of all 20397 mouse genes, where the genomic locations of the promoters are based on RefSeq gene annotations. Evolutionary conservation was used as an additional data sources as explained above and binding specificities for 266 TFs were again taken from TRANSFAC Professional version 10.3. Prior to analyzing the promoter sequences, DNA repeats were found using RepeatMasker [80] and ignored from further analysis. While we mainly focus on general summary results here, the full binding probability results for all mouse TF-gene pairs (20397×266 table) are available on the supplementary web page.

**Figure 14:**
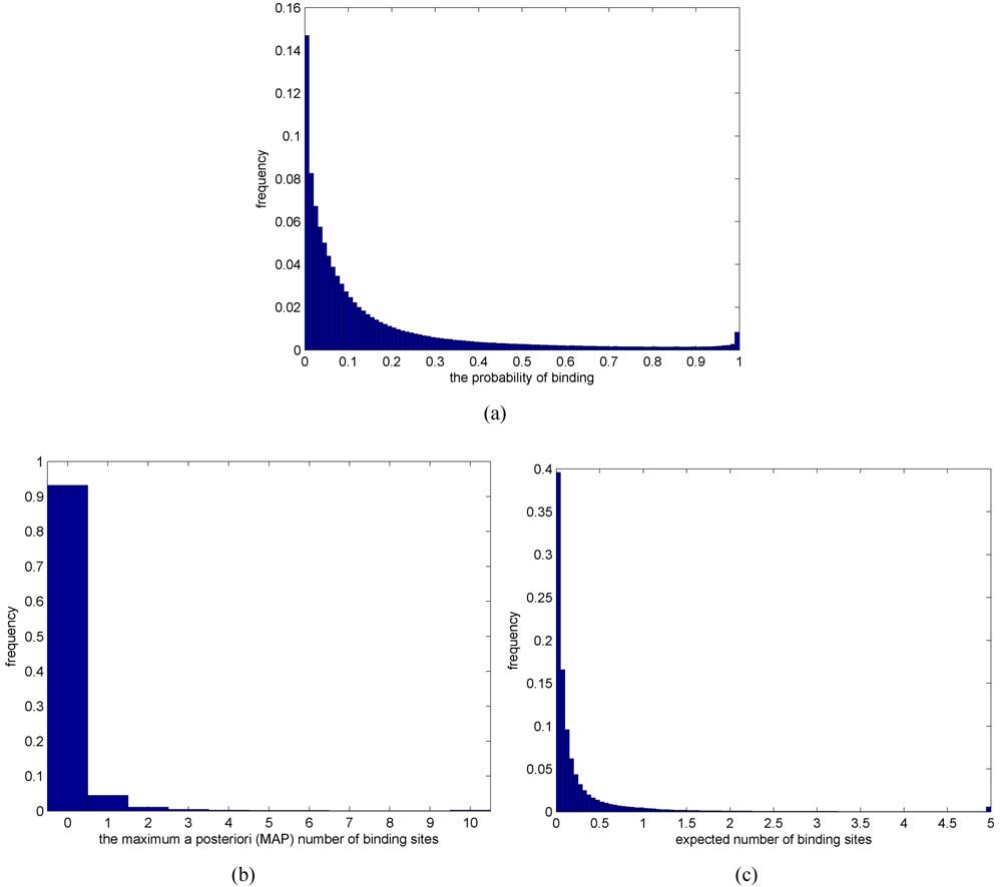
Histogram of (a) the estimated binding probabilities, (b) maximum a posteriori (MAP) number of binding sites, and (c) the expected number of binding sites over all 5.4 million TF-promoter pairs. Histogram frequency at bin value 10 in Figure (b) (resp. value about 5 in Figure (c)) includes all values that exceed 10 (resp. 5).

Figures 14 (a)–(c) show histograms of the estimated binding probabilities, maximum a posteriori (MAP) number of binding sites and the expected number of binding sites, respectively, over all 5.4 million TF-promoter pairs. A histogram of the estimated binding probabilities in Figure 14 (a), for example, shows strong bias towards weak binding probabilities. Similarly, zero binding sites is by far the most frequent case among the MAP number of binding sites (Figure 14 (b)) and the expected number of binding sites are also heavily biased towards small values (Figure 14 (c)). These findings are consistent with the current view that, on average, biological interaction networks, such as transcriptional regulatory networks, are sparsely connected (see [81]). Perhaps more interestingly, the histogram of binding probabilities is clearly bi-modal. The second, smaller peak is located at high binding probabilities, close to the probability value 1. Therefore, the histogram of binding probabilities can, for example, be considered as a mixture of two exponentially decreasing (no binding) and increasing (binding) distributions.

**Figure 15:**
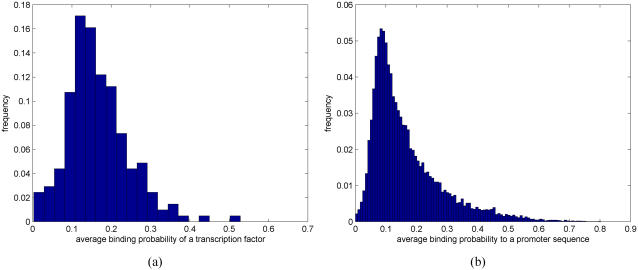
Histogram of the estimated average binding probabilities over (a) different TFs and (b) promoter sequences.

Figures 15 (a) and (b) further summarize the estimated binding probabilities over different TFs and promoter sequences, respectively. Although most TFs have relatively low average binding probabilities (Figure 15 (a)), say between 0 and 0.3, average binding probabilities also possess some degree of variability. For example, the TF that has the highest average binding probability (about 0.52) is the well-known Sp1 protein (see [82]) that has been reported to bind practically everywhere in the human genome [83]. Figure 15 (b) displays the histogram of the average binding probability to a promoter sequence. Most of the average binding probabilities are again relatively small, say between 0 and 0.3, but there are also a few promoter sequences for which the average binding probability is higher. This feature is often referred to as scale-free. Note that by scaling the *x*-axis of Figure 15 (b) by the number of TFs (266) one gets an estimate of the number of regulators per gene.

## Discussion

In our analysis, we primarily focused on estimating the binding probability of a TF to either the whole analyzed promoter or at single base pair resolution (using the Bayesian method). We also introduced an extension for inferring combinatorial regulation. Given the flexibility of our probabilistic (Bayesian) modeling framework, virtually any question can be answered probabilistically within it. For example, Beer and Tavazoie [51] introduced a method for predicting gene expression using positional and combinatorial constraints for local sequence elements. Similar questions can also be answered probabilistically in the proposed framework, e.g., “what is the probability that two TFs, A and B, both have binding sites in a given promoter such that binding site for A is closer to the transcription start site than that of B, binding sites are within 50 base pairs from each other and within 150 base pairs from the transcription start site?” Alternatively, the above type of positional and combinatorial constraints that have been identified in previous studies can be included into the proposed framework via informative prior distributions.

One popular way of analyzing expression data is based on clustering similarly behaving genes together or finding groups of genes that are differentially expressed. The gene sets found are then typically searched for common (either known or unknown) sequence motifs. A potentially very useful extension of our framework will be to develop a method for computing the probability that a set of genes (or a fraction of them) have a binding site for a TF or for a set of TFs.

A number of other possible extensions are also easily included in this probabilistic modeling framework. For example, some proteins interact to form a heterodimer and bind as a complex, in which case the potential binding sites of (all or a subset of) the constituent TFs may be more likely to be physically close to each other. Incorporation of protein-protein interaction databases may help in revealing such mechanisms. Evolutionary conservation was included in the framework by utilizing the conservation scores of an input promoter. One can also simultaneously analyze the corresponding promoter in other organisms to check if they have a binding site for the same TF (in the corresponding location), see [46]. In addition to this, an interesting extension would be to modify the proposed framework to take into account the conservation of TF binding patterns [84]. An alternative future extension is to incorporate probabilistic evolutionary processes within the proposed binding prediction framework (see [6]). Finally, as noted before, our choice of using a PSFM model for binding sites is arbitrary and the same framework can be easily extended to other binding site models as well.

Our final note is devoted to the general distinction between motif discovery and binding site prediction methods. The proposed Bayesian method interprets binding specificities as random variables whose prior parameters define the amount of uncertainty associated with each TF binding model. By gradually forcing all pseudo counts to be equal (unity), i.e., increasing the uncertainty, the Bayesian binding prediction method indeed turns into a pure motif discovery method. Thus, for uncertain binding specificities, the Bayesian method can also be used as a motif discoverer.

Our future work includes developing the framework in the direction of the aforementioned extensions. We are also extending our genome-wide analysis to yeast. Predicting TF binding in yeast is interesting not only because it is the most often considered model organism, but also because yeast has a well-developed nucleosome model [74] and more abundant ChIP-chip data (see [65], [9]).

A central goal in the described computational analysis is accurate TF binding prediction from multiple data sources. However, because TF binding does not necessarily imply transcriptional regulation, it is also important to further extend computational methods to incorporate other, functional data, such as gene expression or protein level (time series) measurements. Statistical inference of transcriptional regulatory networks from a combination of gene expression time series, promoter sequence and binding specificity data has been studied in [85]–[87], [63]. Probabilistic formulation of TF binding prediction is particularly useful in such modeling approaches as it naturally allows a principled fusion of various data sources. In a Bayesian context, it is both intuitive and simple to integrate diverse data sources via informative prior distributions. This is similar with what we proposed for the probabilistic integration of multiple data sources above. Similar ideas have already been introduced for the Bayesian inference of transcriptional regulatory networks from gene expression data where a prior distribution of network models is estimated from promoter sequences and TF binding specificities [63] or from ChIP-chip data [62]. We are currently developing Bayesian learning methods for transcriptional regulatory networks that can make principled statistical inference from diverse sets of data sources, such as the ones already discussed in this work and other functional data.

We have developed a flexible and comprehensive framework for TF binding prediction from multiple data sources. The proposed methods are probabilistic in nature and, thus, directly assess our degree of belief in binding or non-binding in terms of probabilities. Instead of assessing TF binding at each nucleotide location separately, we extended the binding prediction methods to analyze each promoter sequence as a whole. This gives a more complete view of a TF binding to and possibly regulating a target gene. Although we primary focused on answering the question of whether the entire promoter has a binding site for a TF, we also developed a method for computing binding probabilities at each nucleotide position by essentially integrating out other locations in a promoter. Most importantly, the proposed methods can make principled inference from multiple data sources that can include, among others, multiple motif models, evolutionary conservation, regulatory potential, CpG islands, nucleosome positioning, DNase hypersensitive sites and ChIP-chip. Results on our carefully constructed test set demonstrate that principled data fusion can significantly improve the performance of binding prediction methods. Recent technological developments, such as protein binding microarrays, now allow accurate measurements of TF binding specificities to be gathered in a high-throughput fashion. Using accurate binding specificity measurements together with principled TF binding prediction methods can provide a competitive alternative to traditional condition specific ChIP-chip experiments, especially when TF binding prediction incorporates multiple additional data sources. Our genome-wide TF-DNA binding results for mouse indicate relatively sparse connectivity between TFs and their target genes, consistent with previous results. The probabilistic formulation of TF binding prediction is particularly useful for integrating our results as building blocks in other computational methods. To that end, we have also implemented a web tool, ProbTF, which allows users to analyze their own promoter sequences and additional data sources.

## Materials and Methods

The computational methods are implemented in Matlab and will be made available as an open-source library upon publication. The test set, including all sequences and additional information sources, will also be made available on a supplementary web site. A preliminary version of the computational methods presented in Sections ‘Modeling framework’, ‘Likelihood approach: one motif model θ’, and ‘Likelihood approach: multiple motif models Θ’ have been reported in our previous conference article [88].

### Modeling framework

Let *S* = (*s*_1_,…,*s_L_*) denote a single strand of a promoter sequence, where *s_i_*∈{A, C, G, T} and *L* is the length of the sequence. (Generalizations to double stranded DNA sequences are given later on.) Let *Q* denote the number of (hidden) motif instances in sequence *S*. This is one of the key quantities estimated from the data. Further, let *A* denote the (unknown) start positions of non-overlapping motif instances in sequence *S*. For example, if *Q* = *c*, then *A* = {*a*_1_,…,*a_c_*}. Thus, a promoter consists of *c* motif instances and *c*+1 background sequence chunks, some of which can be empty. In the following we assume that *A* always defines start positions for non-overlapping motifs.

Non-binding background sequence locations are modeled by the commonly used *d*th order Markovian background model φ. That is, let
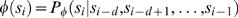
denote the probability of observing nucleotide *s_i_* at the *i*th position of a promoter sequence *S* in the background model φ given *d* previous nucleotides. For simplicity, we assume that for positions *i*≤*d* we have access to *s*_−*d*+1_,…,*s*_0_. We could alternatively define a separate probability distribution for the first *d* nucleotides. The likelihood of the background model, *A* = Ø, is thus 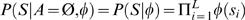.

Motifs are modeled using the standard PSFM model θ which is a product of independent multinomial distributions [21]. Similarly as above, let
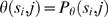
denote the probability of observing nucleotide *s_i_* at the *j*th (*j* = 1,…,ℓ) position of a motif model θ, where ℓ is the length of the motif. Note that 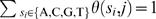 for all *j* and that probabilities for different *j*s are independent. The probability of sequence *S*, given non-overlapping motif positions and motif and background models, is
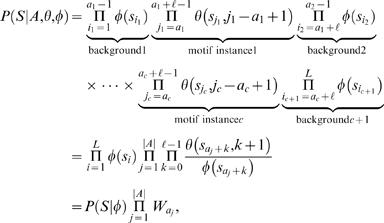
(3)where |*A*| = *Q* = *c* and 
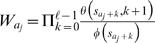
.

### Likelihood approach: one motif model θ

Using Bayes' rule, the probability of *c* motif instances, given the sequence *S*, is (see [42])
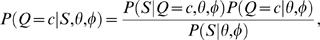
(4)where the normalization factor has the following form
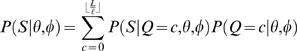
(5)and 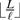 is the maximum number of non-overlapping ℓ-length motifs in an *L*-length sequence. Note that since the sum in Equation (5) has only 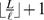 terms (instead of infinitely many) the normalization factor can be computed exactly. The likelihood of sequence *S*, given that it contains *c* motif instances, can be obtained by summing over all possible positions *A* of *c* motif instances [42]

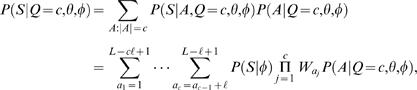
(6)where in the last equality we have used *P*(*S*|*A*,*Q* = *c*,θ,φ) = *P*(*S*|*A*,θ,φ) and Equation (3). The above probabilistic formulation (Equations (4)–(6)) is practically identical to the one proposed by Thijs *et al.* [42].

As in [42], let us assume for now that, for a fixed value of *Q*, the prior over motif positions *A* is uniform and is inversely proportional to the number of different motif positions, i.e., 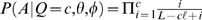. Let *R*(*S*|*Q* = *c*,θ,φ) denote the sum in Equation (6) without the (constant) prior term *P*(*A*|*Q* = *c*,θ,φ). The likelihood in Equation (6) can be computed efficiently using the following recursion
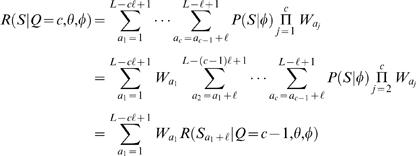
where 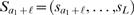 denotes a subsequence of *S* (note that *S*_1_ = *S*). For the prior over the number of motif instances, we use a probability distribution motivated by previous studies [42]

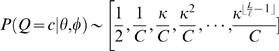
(7)where 
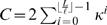
. Since *P*(*Q* = *c*|θ,φ) does not depend on θ or φ we also rewrite *P*(*Q* = *c*|θ,φ) = *P*(*Q* = *c*). One could consider other priors as well, such as ones that depend on the information content of the matrix (see [6]).

The probability that a given TF (defined by θ) binds to a gene having promoter sequence *S*, denoted by θ→*S*, can be computed as
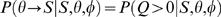
(8)

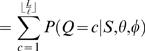
(9)

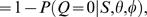
(10)where *P*(*Q* = *c*|*S*,θ,φ) can be obtained using Equations (4)–(7). Assuming a single binding position is sufficient for transcriptional regulation, then the above probability of binding can also be interpreted as a probability of transcriptional regulation. It is straightforward to adopt the above probability *P*(θ→*S*|*S*,θ,φ) for the requirement of having multiple binding sites. Also note that we have the distribution of having any number (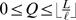) of binding sites from which we can compute, e.g., the expected (mean) and the maximum a posteriori (MAP) number of binding sites.

### Likelihood approach: multiple motif models Θ

A TF can recognize several different types of binding sites and is then characterized by several motif models Θ = (θ^(1)^,…,θ^(*m*)^) each having length ℓ*_i_*. Let π∈{1,…,*m*}*^c^* denote a configuration of motif models from Θ in *A*. That is, π*_i_* specifies the motif model 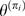 at location *a_i_*. For notational convenience, define
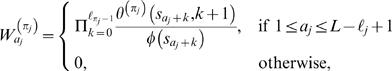
(11)and note that (see also Equation (3))
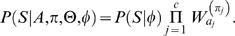
(12)


The probability of *c* motif instances can be obtained using Bayes' rule as in Equations (4)–(5) but θ replaced with Θ. Further, following Equation (6), the likelihood of sequence *S* given *c* motif instances can be obtained by summing over all possible positions and configurations
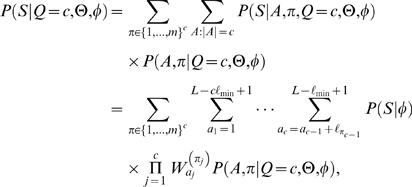
(13)where ℓ_min_ = {ℓ_1_,…,ℓ*_m_*}. Let us again start by assuming a uniform prior over motif positions *A* and configurations π (for each fixed value of *Q*), and let *R*(*S*|*Q* = *c*,Θ,φ) denote the sum in Equation (13) without the (constant) prior term *P*(*A*,π|Q = *c*,Θ,φ). A computationally efficient recursive formula can be written as
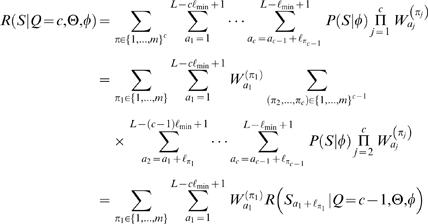
A closed form formula for uniform *P*(*A*,π|*Q* = *c*,Θ,φ) is more difficult to obtain in general, but it can be computed numerically using a similar recursion as the one above.

The prior *P*(*Q* = *c*|Θ,φ) depends now on Θ and thus can be adjusted for multiple motifs. However, it is unrealistic to assume that different motif models (θ^(1)^,…, θ^(*m*)^) are independent. Indeed, it is likely that they are strongly dependent. Therefore, we use the same prior *P*(*Q* = *c*) as in the case of a single motif model as a first approximation.

Let Θ→*S* denote that a TF characterized by Θ binds to a promoter *S*. The probability that at least one of the motif models in Θ has a binding site in *S*, *P*(Θ→*S*|*S*,Θ,φ), can be computed as in Equations (8)–(10) but θ replaced with Θ. Under the same premise as above that a single binding site is sufficient for gene regulation, *P*(Θ→*S*|*S*,Θ,φ) can be interpreted as the probability of regulation.

The above probabilistic modeling framework that incorporates multiple motif models can be viewed as an extension of a framework proposed in [42]. Note that the proposed framework is also similar to hidden Markov models (HMM) that have been proposed previously [36]–[38], [44]. An HMM is defined by motif and background models Θ and φ and the transition probabilities (between the states of the HMM) whereas the modeling framework described herein is built on motif and background models alone, with additional information brought into the computation via the priors *P*(*Q*|Θ,φ) and *P*(*A*,π|*Q*,Θ,φ).

### Bayesian approach

TF binding specificities are derived from experimental data sets, some of which have extremely small sample sizes (as low as five reported binding sequences). PSFM models can therefore contain a considerable amount of uncertainty. Instead of assuming motif models θ to be known exactly, as above, it is useful to take the uncertainty in the motif models themselves into account. This can be done naturally in a Bayesian setting where the parameters/models are considered as random variables. We describe the Bayesian methods directly for the case of multiple motifs. The single motif case can be obtained as a special case by setting *m* = 1 and omitting π.

Using Bayes' rule, the probability of motif positions *A* and configurations π, given the sequence *S*, is
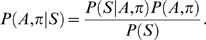
(14)


The marginal likelihood *P*(*S*|*A*,π) is obtained by integrating over parameters
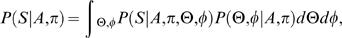
where *P*(*S*|*A*,π,Θ,φ) is the same product of multinomial distributions as in Equation (12) and *P*(Θ,φ|*A*,π) = *P*(Θ,φ)defines a prior distribution for the parameters.

TF-DNA binding databases typically provide information in the form of “the number of times a TF has been observed to bind a given sequence.” These sequences are also aligned and aligned counts are summarized in position specific weight matrices (TRANSFAC, JASPAR), which we denote as α*_ij_* ^(*k*)^. Similar counts can also be obtained for the background model (denoted by α*_ij_* ^(0)^) from genomic sequences that do not contain (known) binding sites. Therefore, it is natural to use a Dirichlet prior for the parameters, which is defined by so-called pseudo-counts.

Let us rewrite the motif model parameters (independent multinomial distributions) for now as θ^(*k*)^(*i*, *j*) = θ*_ij_* ^(*k*)^ which again defines the probability of seeing nucleotide *i*∈{A, C, G, T} at the *j*th (1≤*j*≤ℓ*_k_*) position in the *k*th (1≤*k*≤*m*) motif model. Denote θ*_j_* ^(*k*)^ = {θ*_ij_* ^(*k*)^|*i*∈{A, C, G, T}}. The Dirichlet prior for each θ*_j_* ^(*k*)^ with hyperparameters α*_ij_* ^(*k*)^ is defined as
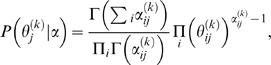
where θ*_ij_* ^(*k*)^≥0, ∑*_i_*θ*_ij_* ^(*k*)^ = 1, α*_ij_* ^(*k*)^>0, and Γ(·) is the Gamma function. Priors for different *j* and *k* are assumed to be independent. The Dirichlet prior for the background model is defined similarly.

The Dirichlet prior is also a conjugate prior for multinomials. Consequently, the marginal likelihood has a closed-form solution. Let *N_ij_* ^(*k*)^ denote the number of times nucleotide *i* is observed at the *j*th position in the *k*th motif model given *S*, *A* and π. Denote α*_j_* ^(*k*)^ = ∑*_i_*α*_ij_* ^(*k*)^ and *N_j_* ^(*k*)^ = ∑*_i_N_ij_* ^(*k*)^. The marginal likelihood can be written as
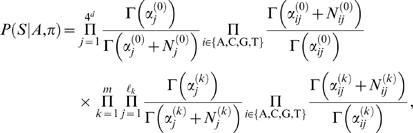
(15)where the first (resp. the second) part corresponds to the background model (resp. *m* motif models).

To keep likelihood-based and Bayesian approaches comparable, we use the same prior here, i.e.
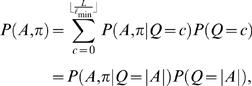
(16)where *P*(*A*,π|*Q* = |*A*|) and *P*(*Q* = |*A*|) are as in Equations (13) and (7), respectively. Note that we write *P*(*A*,π|*Q* = |*A*|) instead of *P*(*A*,π|*Q* = |*A*|, Θ,φ) because the (uniform) prior depends only on the widths of the motif models, ℓ*_i_*, and not on the actual parameters Θ or φ.

Because some of the motif models are remarkably diffuse (computed from only a few example sequences), we do not use the PSWMs as pseudo counts directly. We instead use a version that incorporates a so-called prior strength term *M*, i.e.,
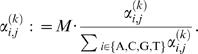
(17)


This prevents a (single) sequence to have too strong of an influence on the posterior parameter values. For simplicity, we use the same prior strength for all the motif models, although this does not need to be the case in general. In addition, we add a small number (one) to each α*_i_*_,*j*_^(*k*)^ to prevent zero entries. Finally, to preserve comparability between likelihood and Bayesian approaches, the normalized motif models for the likelihood based approach are computed from the recomputed pseudo-counts used in the Bayesian estimation, i.e.,
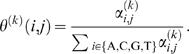
(18)


Recall that in the Bayesian framework, the mean of θ^(*k*)^(*i*, *j*) relative to the prior distribution *P*(θ*_j_* ^(*k*)^|α) is equal to the quantity in Equation (18).

### MCMC estimation for Bayesian inference

Unfortunately, there is no efficient recursive formula to compute the probabilities *P*(*A*,π|*S*) for all *A* and π. However, one can solve the problem by using stochastic estimation methods. Here we propose to sample positions *A* and configurations π directly from the posterior *P*(*A*,π|*S*) using Markov chain Monte Carlo (MCMC). We develop a Metropolis-Hastings (MH) algorithm for this purpose. For an introduction to MCMC methods, see [89].

The MH algorithm is completely specified by a proposal distribution *G*(*A*′,π′|*A*,π) which proposes new pairs (*A*′,π′) given the current (*A*,π). We define *G* as follows.

- Motif addition with probability *p*: for a uniformly chosen motif model θ^(*i*)^∈Θ, propose a new, non-occupied/non-overlapping motif position uniformly randomly (if a free location exists).
- Motif deletion with probability 1−*p*: delete an existing motif uniformly randomly (if a motif exists).

We use *p* = 0.5. The proposed pair (*A*′,π′) is then accepted with probability
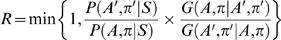
which satisfies the detailed balance condition. Convergence in distribution to the desired posterior (in the limit of infinitely many samples) is guaranteed if, in addition to satisfying the detailed balance, the resulting chain is also irreducible and aperiodic. Irreducibility of the chain follows from the fact that any pair (*A*′,π′) can be reached from any pair (*A*,π) by repeatedly adding or deleting one motif at a time and each step has a positive selection probability. Aperiodicity of the chain can be seen similarly. For example, the probability that the chain first deletes all *c* = |*A*| motifs, then stays at *A* = Ø any number of steps (by trying to delete a motif), and finally adds the same *c* motifs, is non-zero. In other words, the probability of moving from (*A*,π) back to (*A*,π) in 2*c* or more steps is non-zero. Therefore, the period (the greatest common factor of integers that include at least {2*c*, 2*c*+1,…}) for all the states (*A*,π) is 1 and the chain is aperiodic. Note that computing the above Bayes factor can be done very efficiently because only a single motif is added or deleted at a time. In particular, only two parts of the marginal likelihoods are different, corresponding to the motif that is added/deleted and the background chunk that is deleted/added. For integer-valued pseudo-counts, the computation of the Bayes factor reduces even further because Γ(*n*) = (*n*−1)!. After a proper burn-in period *B*, a dependent sample ((*A* ^(*B*+1)^, π^(*B*+1)^), (*A* ^(*B*+2)^, π^(*B*+2)^), …, (*A* ^(*B*+*N*)^, π^(*B*+*N*)^)) is collected.

Although the chain is ergodic as shown above, it is important to monitor convergence of the MCMC algorithm for finite samples to guarantee the desired output. Bayesian inference in this case can be considered as a model selection problem where the model space consists of all valid pairs (*A*,π). Although the model space is discrete and finite, standard convergence diagnostics over the full model space are difficult to apply in practice. Better suited diagnostic methods are the ones that are specifically developed for model selection problems, such as the ones in the context of reversible jump MCMC methods (see [90]). A general strategy is to reduce the model space and monitor the convergence in a lower dimensional space. Here we consider a method that compares the marginal probabilities of having 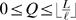 binding sites, *P*(*Q* = *c*|*S*) = ∑_|*A*| = *c*_ ∑_π_ *P*(*A*,π|*S*), from two independent chains (see [91]). Note that *P*(*Q* = *c*|*S*) is exactly the distribution that we are interested in when assessing TF binding. As for the convergence diagnostic, we use a heuristic that reports two chains as having converged if the *L*_1_-distance between two independent estimates of *P*(*Q*|*S*) is within an accepted error threshold (we use 0.025). We could also use a formal hypothesis testing (e.g. chi-squared or Kolmogorov-Smirnov) for assessing lack of convergence by sub-sampling the two chains to get (approximately) independent samples [90]. The less involved heuristic seems to serve our purposes. We use *B* = 5·10^5^ for the burn-in and *N* = *i*·*B* for the sample size, where index *i* = 1, 2,… is increased until the chain pair passes the convergence diagnostic.

As above, the quantities of interest include the probability of having at least one binding site for at least one of the motif models, denoted as Θ→*S* (or, conversely, having no binding sites),
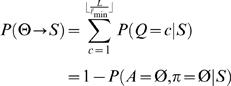
and the posterior probability of having exactly *Q* = *c* binding sites, *P*(*Q* = *c*|*S*). These quantities can be estimated directly from the chain
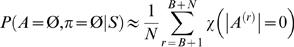
and
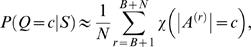
where χ is the indicator function. The sample averages converge to the true posterior probabilities almost surely. The final estimates are obtained by averaging estimates from two independent chains that pass the convergence diagnostic.

### Combinatorial regulation

From the point of view of modeling transcriptional regulatory networks, it is also important to study combinatorial regulation by several TFs Ω = (Θ^(1)^,…, Θ^(*p*)^), each with a set of motif models
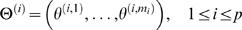
A key quantity then is the probability that each different TF has at least one binding site (for at least one of their motif models). Let the configurations now be defined as
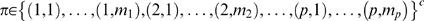
and define a set
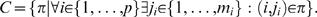
(19)


In other words, the set *C* says that every TF is presented with some motif in every configuration π. Note that Equation (19) implies that if π∈*C* then |π| = *c*≥*p*. The probability of *p* TFs binding a promoter *S* jointly can be written as
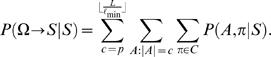



Probabilities *P*(*A*,π|*S*) are computed as in Equations (14–16) except that *m* in Equation (15) is replaced with *m*_1_+…+*m_p_* and the prior *P*(*A*,π) defined in Equation (16) is adjusted for several TFs. Let *c_i_*≥0 denote the number of binding sites for the *i*th TF. Assuming that the number of binding sites for different TFs are independent, then one can model the joint number of binding sites, *Q*_Ω_, as
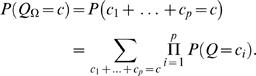



There is no efficient formula to compute *P*(Ω→*S*|*S*), or even the analogous likelihood based quantity, so we only formulate this in the Bayesian context and solve it with MCMC sampling. The same MH algorithm as above can be applied except that a motif θ^(*i*,*j*)^ is now added uniformly randomly from a list of motif sets Ω. Finally, *P*(Ω→*S*|*S*) can be directly estimated from a chain as
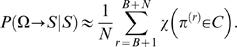
(20)


We also consider a naive (likelihood-based) approximation that estimates the probability of combinatorial regulation by the product of individual binding probabilities, i.e.,
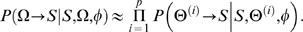
(21)


### Combining multiple information sources

TF binding predictions can be significantly improved by incorporating multiple additional data sources, such as evolutionary conservation, regulatory potential, CpG islands, nucleosome positioning, DNase hypersensitive sites or ChIP-chip, into our probabilistic inference framework. Let *D* denote a single additional data source that is indicative of functional binding sites. The data is assumed to be in the form *D* = (*P*(1),…, *P*(*L*)), where *P*(*i*) is the probability that the *i*th base pair location has one of the above mentioned properties (is conserved, belongs to a regulatory region, has a low nucleosome occupancy, etc.). Here we explain how such additional information can be used in a principled way to significantly improve TF binding inference.

We model the probability of *S* and *D* given *A*, π, Θ and φ as
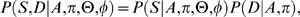
where we assume that *S* and *D* are conditionally independent and that the probability of *D* does not depend on the motif and background models. Let *I* = {1,…, *L*} denote base pair indices of a promoter and 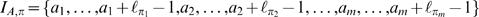 be indices of binding sites specified by *A* and π. The data *D* can then be modeled as
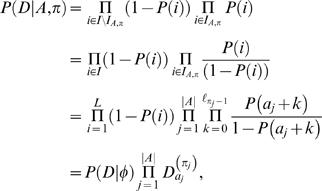
(22)where 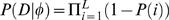 and
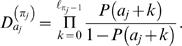



The factorization in Equation (22) is useful as it allows us to write *P*(*S*, *D*|*A*,π,Θ,φ) in the following compact form
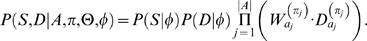



In particular, note that in the likelihood based approach the same efficient recursive formula as in ‘Likelihood approach: multiple motif models Θ’ Section can be applied to compute *P*(*S*, *D*|*Q* = *c*,Θ,φ).

In a Bayesian setting, data fusion can be performed similarly
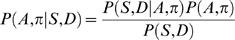
(23)

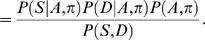
(24)


If we have several additional data sources *D_k_* = (*P_k_*(1),…, *P_k_*(*L*)), 1≤*k*≤*N_D_*, then we propose to combine them directly using
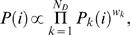
where *w_k_*≥0 and 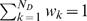.

Conceptually, the probability *P*(*D*|*A*,π) can be viewed as a positional prior for binding sites. But we do not combine *P*(*D*|*A*,π) with *P*(*A*,π) since all aforementioned data sources (probabilities *P*(*i*)) depend on the sequence *S* as well.

In practice, we do not use the above probabilities directly but a scaled version of them. For the *k*th additional data set we scale the original probabilities between [*d_k_*, 1−*d_k_*], where 0≤*d_k_*≤0.5. We resort to fixed parameter values for *w_k_*s and *d_k_*s here because the estimated binding probabilities are not sensitive to small deviations in the value of *w_k_* or *d_k_* However, it is also possible to develop a full Bayesian treatment by introducing priors over *w_k_*s and *d_k_*s. Our simplified strategy also improves computational efficiency and makes it possible to use the standard MCMC instead of more computationally demanding trans-dimensional MCMC methods.

### Single vs. both strands

So far we have only considered using a single, either forward or reverse, strand for inferring TF binding. Extending the above methods to double-stranded DNA is straightforward. Let a promoter be denoted now as *S* = (*S*′,*S*″), where *S*′ and *S*″ are the forward and complementary reverse strands. Let us first assume that binding events on separate strands are independent of each other. In the case of double-stranded DNA, we need to compute the probability of an event Θ→*S*′ or Θ→*S*″. Let us denote *P*(Θ→*S*′|*S*,Θ,φ) = *P_S_*_′_ and *P*(Θ→*S*″|*S*,Θ,φ) = *P_S_*_″_. Now
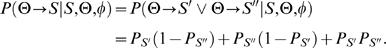



Due to the OR-type of event (Θ→*S*′ or Θ→*S*″), the effective prior probability of having zero binding sites decreases. We account for this bias by changing *P*(*Q* = 0|Θ,φ) from 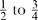. The above formulation can also make use of the correlation between *P_S_*_′_ and *P_S_*_″_. A similar extension works for the Bayesian case as well with the exception that *S*′ and *S*″ should not be analyzed separately even if binding on the two strands is assumed to be independent. Joint analysis of *S*′ and *S*″ is easily implemented using the same MCMC/MH algorithm as above. The only difference is that now the model should include separate start positions *A* = (*A*′,*A*″) and configurations π = (π′,π″) for the two strands. This approach also allows incorporating additional constraints, such as binding sites on two strands are less likely to occur at the same position due to physical space constraints.

### Binding probabilities at single nucleotide resolution

Our computational methods are primarily designed to answer the question of whether the whole promoter has a binding site for a given TF. However, it is also useful to be able to infer binding probabilities at higher, single nucleotide, resolution. A motif position and configuration pair (*A*,π) contains a binding site (start position) at the *i*th nucleotide if *i*∈*A*. In the Bayesian context, the probability of a binding site at the *i*th location can be expressed and estimated simply as
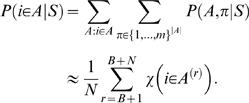



### Comparison with other methods

We compare our proposed probabilistic method with traditional promoter scanning [21]–[23], [26], [28] and with a method that assesses the probability of binding [63]. Traditional scanning methods output a significance value for each position in a promoter. It is common practice to use the smallest *p*-value over a promoter as a measure of binding to the whole promoter, which we also use here. We use the same approach to adopt traditional promoter scanning to handle multiple motif models and report the smallest *p*-value over the multiple motif models. The same approach to handle multiple motif models is used with the method from [63]. Significance values of the traditional scanning method (as well as intermediate quantities in [63]) are computed relative to the null distribution derived from the negative promoter sequences.

### Data

The data set consists of a merger of annotated TF binding sites for mouse from the ABS [35] and ORegAnno [34] databases. In total there are 47 annotated promoter sequences. The sequences have sets of length of around 500 nucleotides (ABS) and 2K nucleotides (ORegAnno). The TF binding sites are mapped onto the mm8 (February 2006) mouse assembly.

The promoters are generally upstream of the genes that they are associated with. However, some regions stretch over the first exon into intronic regions. Therefore, some promoters have exons in their sequence. There were around a dozen overlapping promoters between the two databases. The TF binding sites for these promoters were merged onto the longer 2K ORegAnno sequences. The data set also includes 250 upstream, non-coding sequences that can be used for generating background models and statistics.

The test set used in our simulations consists of 47 promoter sequences, each having a varying number of annotated binding sites (*positive sequences*), and 250 promoter sequences that have no reported binding sites (*negative sequences*). Additional data sources (evolutionary conservation [72], regulatory potential [73], nucleosome location [74], and CpG islands) are available for all the positive sequences. Evolutionary conservation, regulatory potential and CpG island data are downloaded from the UCSC genome browser [33] and nucleosome location predictions are obtained using the method and software from [74]. After removing unknown/unmatched TFs and TFs for which we do not have prior binding specificities in TRANSFAC, we are left with 70 unique TF-promoter pairs in the *positive set*, which include 23 unique TFs. As for the *negative set*, we use all possible pairs of the 23 unique TFs and the 47 promoter sequences that are not in the positive set. We further filter the negative set and ignore those TF-promoter pairs where the TF belongs to the same family as a TF in the positive set (for the same promoter) since their binding specificities are defined by largely overlapping TRANSFAC motif models. For example, Mef2c has an annotated binding site in the Des promoter. Consequently, all other “Mef-family”-Des pairs are ignored from the negative set. This filtering reduces the negative set from all possible 23×47−70 = 1011 pairs to 952. Because all the annotated binding sites for each TF are on the same strand of DNA, we first use only the strand containing the annotated sites. This also doubles the size of the negative set. Because the direction of transcription can be unknown in general, we also extend the analysis to cover both strands. In some simulations we use only 4 randomly chosen TF-promoter pairs for each promoter from the negative set, resulting in 47×4 = 188 unique TF-promoter pairs in the *reduced negative set*.

For all the simulations, we use (scaled) motif models from TRANSFAC. Parameters of the Markovian background models (model orders 0,1,…, 4 are tested) are estimated from the 250 negative sequences (both strands).

## Supporting Information

Text S1 (0.03 MB DOC)

Figure S1 ROC curves for the likelihood-based probabilistic method (red), traditional scanning (blue), and a probabilistic scanning-based method that outputs a probability of binding (green) for the case where promoter sequence lengths have not been made equal. Background model order is (a) d = 0 and (b) d = 1.
(0.49 MB TIF)

Figure S2 ROC curves for the likelihood-based method (blue) when combined with a single additional information source: regulatory potential (red), and evolutionary conservation (green). Solid graphs (resp. dashed graphs) correspond to the optimized parameters (resp. results obtained with stratified cross-validation).
(0.33 MB TIF)

Figure S3 ROC curves for the traditional scanning (green), traditional scanning combined with thresholded conservation information (blue), probabilistic method combined with conservation information (red), and probabilistic method (cyan) for the case where promoter sequence lengths have not been made equal.
(0.34 MB TIF)

Figure S4 ROC curves for the likelihood-based method (blue) when combined with a single additional information source: evolutionary conservation (green) and regulatory potential (red). Promoter sequences that are used to train the regulatory potential method and that also overlap with our test set have been removed.
(0.33 MB TIF)
