# Supplementary material for: Probabilistic Inference of Transcription Factor Binding from Multiple Data Sources
Source: PLoS One. 2008 Mar 26;3(3):e1820. doi: 10.1371/journal.pone.0001820 (PMC2268002; doi:10.1371/journal.pone.0001820)
Supplement: Text S1 — (0.03 MB DOC) [file pone.0001820.s001.doc]

# Supplemental Material Text S1

Figure S1 shows ROC curves for the comparison of our basic likelihood method with traditional promoter scanning (see [21-24]) and a probabilistic scanning-based method that assesses the probability of binding [63].

Figure S2 shows ROC curves for the likelihood-based method when combined with a single additional information source. Results shown with the solid graphs are obtained by maximizing the AUC measure whereas the dashed graphs are obtained using the stratified cross-validation. For example, for evolutionary conservation, (green) the two graphs, solid green and dashed green, are virtually identical.

Figure S3 shows ROC curves for the comparison of traditional scanning, traditional scanning combined with thresholded conservation information, probabilistic method combined with conservation information, and probabilistic method for the case where promoter sequence lengths have not been made equal.

As noted above, the method to compute regulatory potential scores is discriminatory and requires a set of neutral and regulatory sequences as training data. ESPERR [73] is trained on a set of human genes. This training set is expanded by mapping the set of human genes to orthologous mouse genes, amongst others. This set of mouse genes partly overlaps with our test set. To test whether this overlap introduces any bias, we performed the same human to mouse mapping and removed the overlapping genes from our test set. Although [73] report good generalization properties for regulatory potential, using this smaller test set we can circumvent any possible bias that is caused by over-fitting regulatory potential to the given training data. The obtained ROC curves are shown in Figure S4. Although ROC curves in Figure S4 are overall little lower, the relative performance between different methods remains unchanged. Thus, combining our TF binding inference with ESPERR and applying it to our whole test set does not introduce any bias.
