# Supplementary figures and images for: Probabilistic Inference of Transcription Factor Binding from Multiple Data Sources
Source: PLoS One. 2008 Mar 26;3(3):e1820. doi: 10.1371/journal.pone.0001820 (PMC2268002; doi:10.1371/journal.pone.0001820)

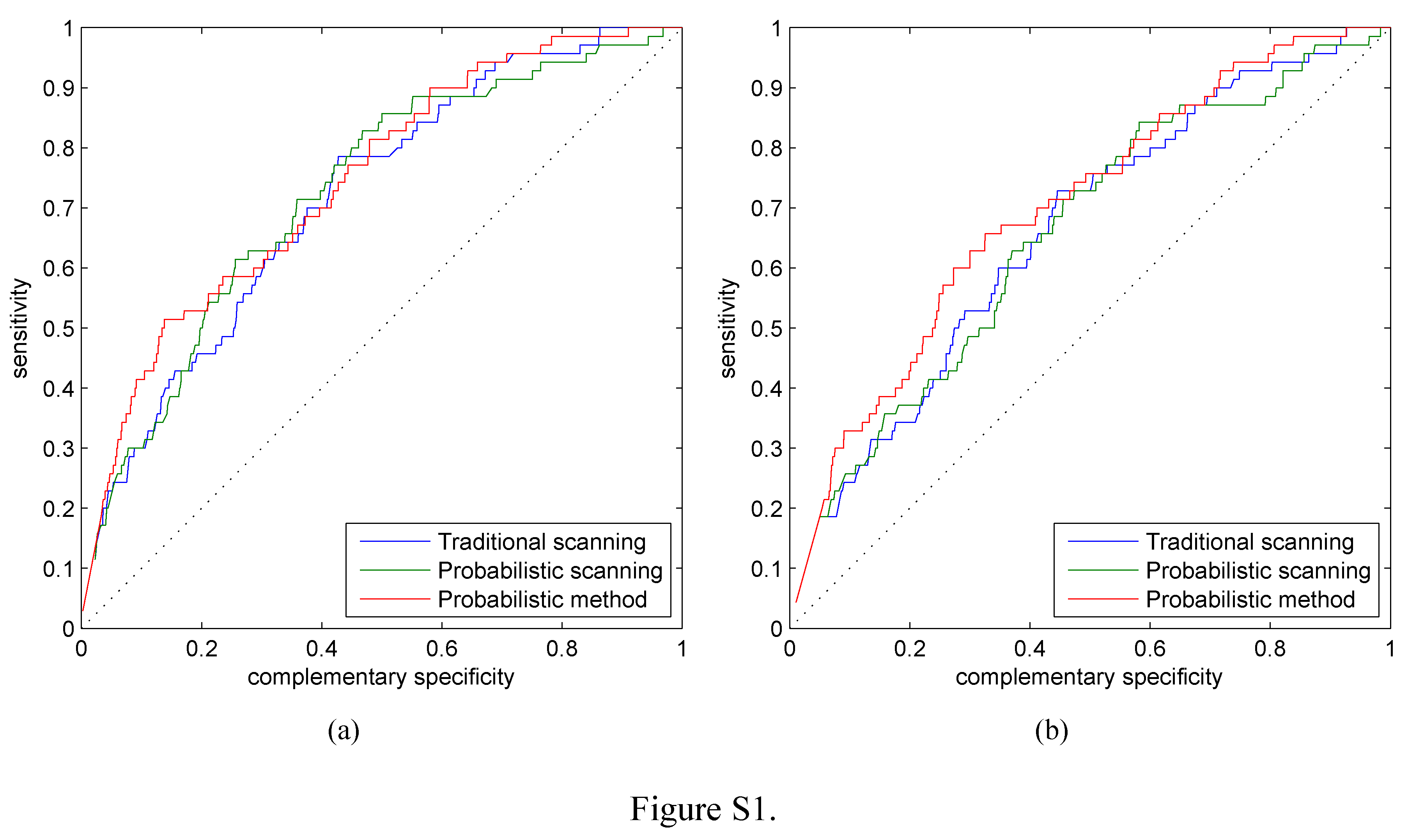

Supplement: Figure S1 — ROC curves for the likelihood-based probabilistic method (red), traditional scanning (blue), and a probabilistic scanning-based method that outputs a probability of binding (green) for the case where promoter sequence lengths have not been made equal. Background model order is (a) d = 0 and (b) d = 1. (0.49 MB TIF) [file pone.0001820.s002.tif]

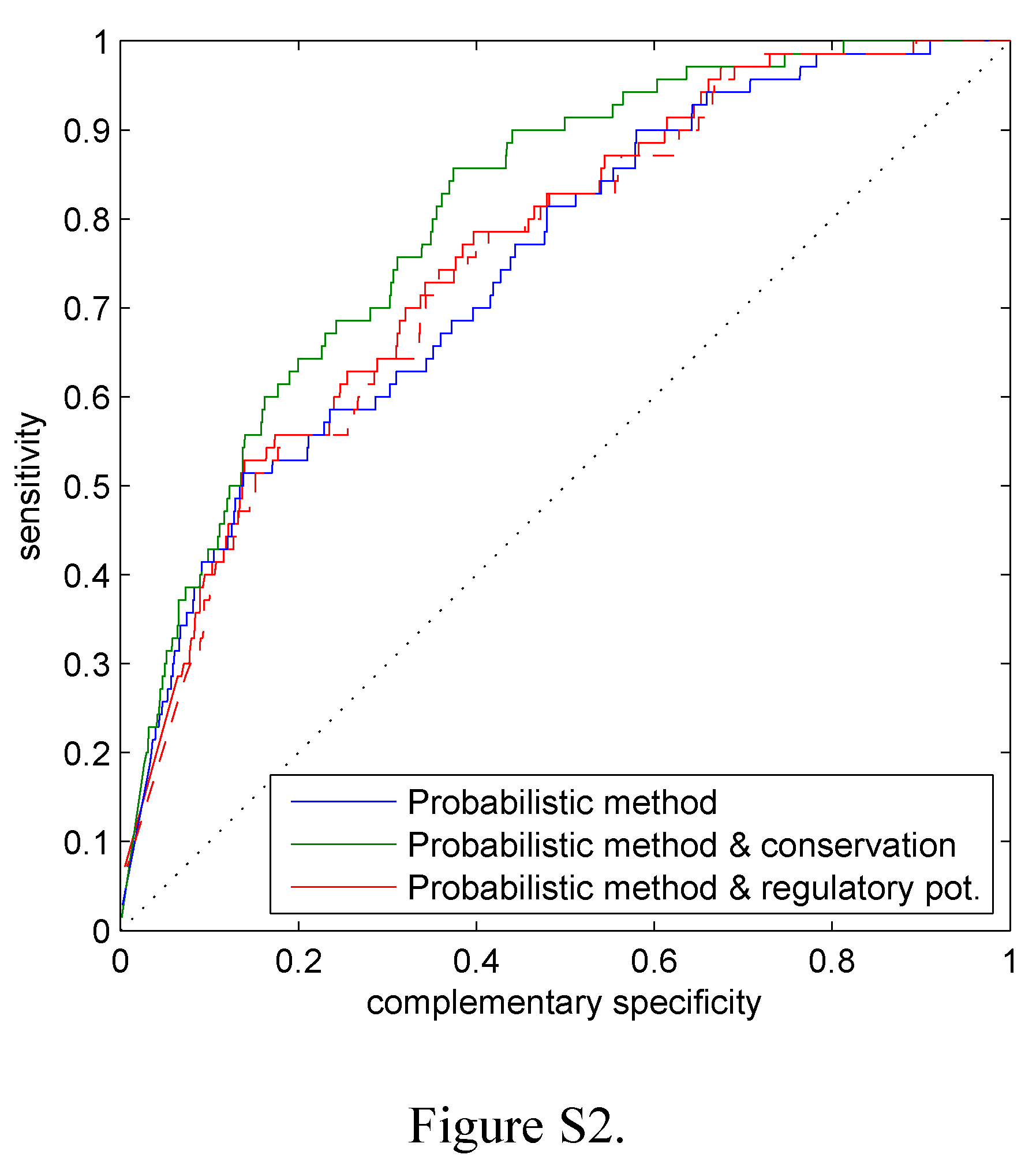

Supplement: Figure S2 — ROC curves for the likelihood-based method (blue) when combined with a single additional information source: regulatory potential (red), and evolutionary conservation (green). Solid graphs (resp. dashed graphs) correspond to the optimized parameters (resp. results obtained with stratified cross-validation). (0.33 MB TIF) [file pone.0001820.s003.tif]

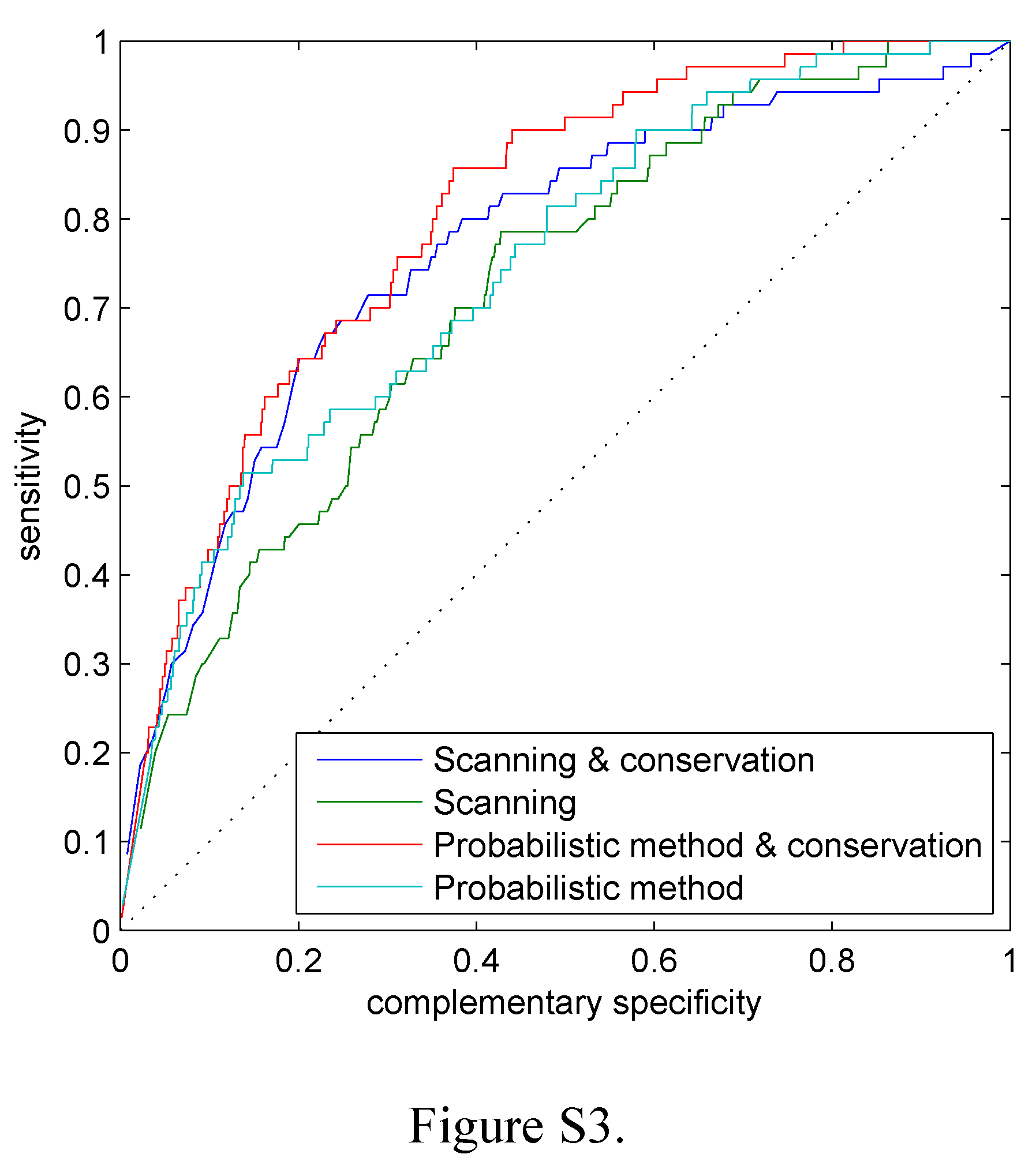

Supplement: Figure S3 — ROC curves for the traditional scanning (green), traditional scanning combined with thresholded conservation information (blue), probabilistic method combined with conservation information (red), and probabilistic method (cyan) for the case where promoter sequence lengths have not been made equal. (0.34 MB TIF) [file pone.0001820.s004.tif]

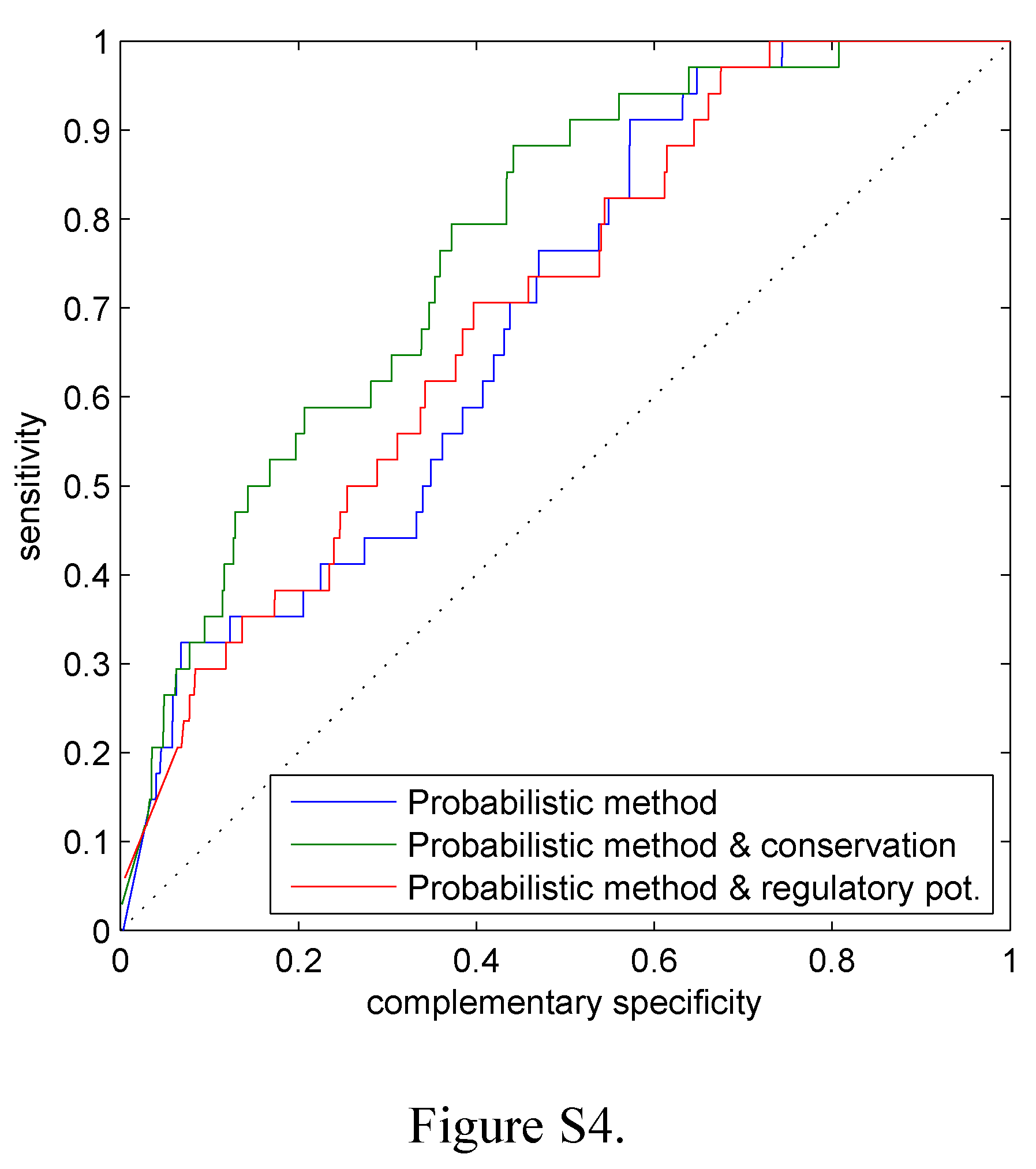

Supplement: Figure S4 — ROC curves for the likelihood-based method (blue) when combined with a single additional information source: evolutionary conservation (green) and regulatory potential (red). Promoter sequences that are used to train the regulatory potential method and that also overlap with our test set have been removed. (0.33 MB TIF) [file pone.0001820.s005.tif]
